# *ZmTH1* Is Vital for Healthy Plant Growth and Promotes Cold/Drought Tolerance by Regulating Thiamin Diphosphate‐Dependent Metabolisms in Maize

**DOI:** 10.1111/pbi.70400

**Published:** 2025-10-13

**Authors:** Tengfei Zhang, Jie Zang, Boming Yang, Qiuxia Wang, Jijun Yan, Peiyong Xin, Jinfang Chu, Huabang Chen, Zhaogui Zhang

**Affiliations:** ^1^ State Key Laboratory of Seed Innovation, Institute of Genetics and Developmental Biology Chinese Academy of Sciences Beijing China; ^2^ University of the Chinese Academy of Sciences Beijing China; ^3^ National Centre for Plant Gene Research (Beijing), Institute of Genetics and Developmental Biology, Chinese Academy of Sciences Beijing China

**Keywords:** maize, TDP‐dependent enzyme, vitamin B1, *ZmTH1*, *ZmTMPS1*

## Abstract

Vitamin B1 (VB1) plays a crucial role in sustaining plant health and enabling adaptive responses to environmental stress. The complex maize genome implies a sophisticated VB1 synthesis pathway, with the mechanisms by which VB1 benefits plants remaining elusive. Here, we identified two VB1 biosynthetic genes, *THIAMINE REQUIRING 1* (*ZmTH1*) and its paralog *THIAMINE MONOPHOSPHATE SYNTHASE 1 (ZmTMPS1*), from a natural mutant *pale leaf and depauperate growth 1* (*pldg1*). We elucidated their specific roles in regulating multiple thiamin diphosphate (TDP)‐dependent metabolic pathways and their effects on plant growth and stress tolerance. *ZmTH1* encodes a chloroplast‐localised, bifunctional enzyme comprising phosphomethylpyrimidine kinase (HMPP‐K) and thiamine monophosphate synthase (TMP‐S) domains. Functional dissection revealed that these domains functioned synergistically, with disruption of one domain significantly attenuating the other, although both can function independently. A frameshift mutation in *ZmTH1* (*Zmth1*) resulted in reduced biosynthesis of VB1, TMP and TDP. Consequently, the activity of TDP‐dependent enzymes was impaired, disrupting multiple TDP‐dependent metabolic pathways. Additionally, *ZmTMPS1*, localised to the cytosol and nucleus, exhibited limited TMP‐S activity that partially compensated for *ZmTH1* mutation in *pldg1* but cannot fully restore VB1 levels. Overexpression of *ZmTH1* or exogenous VB1 application enhanced maize seedling tolerance to cold and drought stresses by increasing TDP‐dependent enzyme activity. These findings advance the understanding of VB1 metabolism in maize and provide genetic targets for improving stress resilience and crop performance.

## 1 Introduction

Thiamin (Vitamin B1, VB1), an essential micronutrient in organisms, participates in key energy metabolisms through its bioactive form thiamin diphosphate (TDP, also known as thiamin pyrophosphate, TPP). TDP serves as a cofactor for enzymes, including α‐ketoglutarate dehydrogenase (α‐KGDH) (Bunik and Fernie 2009), transketolase (TK) (Fiedler et al. 2002), pyruvate dehydrogenase (PDH) (Patel et al. 2014), pyruvate decarboxylase (PDC) (Meyer et al. 2011), 1‐deoxy‐D‐xylulose‐5‐phosphate synthase (DXS) (Zhao et al. 2013), acetolactate synthase (ALS), branched‐chain keto‐acid dehydrogenase (BCKDH) (Binder 2010). These enzymes drive the tricarboxylic acid (TCA) cycle, Calvin cycle, pentose phosphate pathway (PPP), pyruvate metabolism, the methylerythritol phosphate (MEP) pathway for isoprenoid synthesis and the synthesis and degradation of branched‐chain amino acids (BCAAs) (Goyer 2010; Strobbe and Van Der Straeten 2018; Liu et al. 2022). Mutations in VB1 biosynthetic genes in plants result in chlorosis and, in severe cases, plant death (Papini‐Terzi et al. 2003; Ajjawi, Rodriguez Milla, et al. 2007; Ajjawi, Tsegaye, and Shintani 2007; Raschke et al. 2007; Kong et al. 2008; Woodward et al. 2010; Mimura et al. 2016; Hsieh et al. 2017, 2021; Feng et al. 2019; Nie et al. 2022).

VB1 biosynthesis has been extensively studied in *Arabidopsis* and rice (Liu et al. 2022). VB1 comprises a thiazole moiety, hydroxyethylthiazole phosphate (HET‐P) and a pyrimidine moiety, hydroxymethylpyrimidine pyrophosphate (HMP‐PP). HET‐P is synthesised from glycine and nicotinamide adenine dinucleotide through the action of HET‐P synthase THI1, possibly assisted by a NUDIX enzyme (Machado et al. 1996; Chatterjee et al. 2007; Goyer et al. 2013). HMP‐P is generated from aminoimidazole ribonucleotides through the catalysis of HMP‐P synthase THIC (Lawhorn et al. 2004; Raschke et al. 2007; Kong et al. 2008). Subsequently, HMP‐P kinase/thiamine monophosphate (TMP) synthase TH1 phosphorylates HMP‐P to HMP‐PP and catalyses the condensation of HMP‐PP with HET‐P to yield TMP (Kim et al. 1998; Ajjawi, Tsegaye, and Shintani 2007; Nie et al. 2022). TMP, synthesised in the chloroplast, is dephosphorylated by TMP phosphatase TH2 in the cytosol, mitochondria and nucleus to generate VB1 (Mimura et al. 2016; Hsieh et al. 2017, 2021, 2022). Finally, thiamine pyrophosphokinase (TPK) phosphorylates VB1 to TDP in the cytosol (Ajjawi, Rodriguez Milla, et al. 2007).

Additionally, VB1 contributes to both biotic and abiotic stress responses. Under abiotic stress conditions, increased levels of VB1, TMP and TDP, elevated expression of *THI1*, *THIC*, *TH1*, *TPK* and enhanced enzyme activity of TH2, TPK and TK demonstrate the vital role of VB1 in stress responses. External VB1 application further strengthens plant stress tolerance (Ribeiro et al. 2005; Rapala‐Kozik et al. 2008, 2012; Tunc‐Ozdemir et al. 2009; Li et al. 2022). Regarding biotic stress, VB1 application reduces symptoms in infected plants, presumably by inducing systemic acquired resistance through upregulation of defence genes, thereby enhancing disease resistance (Ahn et al. 2005, 2007; Wang et al. 2006; Bahuguna et al. 2012; Li et al. 2022). Furthermore, *THI1* contributes to drought resistance in *Arabidopsis* and mosaic virus resistance in wheat (Li et al. 2016; Yang et al. 2023). Recent metabolic engineering efforts have focused on elevating plant VB1 content to enhance both nutritional value and stress resistance (Dong et al. 2015, 2016; Strobbe et al. 2021a, 2021b; Chung et al. 2024; Fitzpatrick et al. 2024).

Despite these advances, the VB1 biosynthetic pathways in maize remain poorly understood. The complexity of the maize genome suggests a potentially more intricate biosynthetic architecture than in *Arabidopsis* or rice, and the mechanisms through which VB1 enhances plant growth and stress tolerance remain unclear. In this study, we cloned and validated the functions of key maize VB1 biosynthetic genes: *ZmTH1* and its paralog *ZmTMPS1*. We demonstrated the functional independence of the two domains HMP‐P kinase (HMPP‐K) and TMP synthase (TMP‐S) in *ZmTH1*, identified the reduced HMPP‐K activity and absence of TMP‐S activity in the *pldg1* mutant, determined the limited TMP‐S activity of *ZmTMPS1*, and elucidated the collaborative roles of *ZmTH1* and *ZmTMPS1* in maize VB1 synthesis and growth. Dysfunction of *ZmTH1* decreased VB1 synthesis and consequently TDP‐dependent enzyme activity, disrupting metabolic pathways involving TDP‐dependent enzymes and broadly affecting plant growth. This mechanism may explain the symptoms observed in various plants with mutated VB1 genes. *ZmTH1* overexpression or VB1 supplementation improved TMP and VB1 levels and TDP‐dependent enzyme activity and enhanced cold and drought tolerance in maize seedlings. Additionally, the lethal albino phenotype in *ZmTH1* knockout lines resulted directly from MEP pathway disruption. *ZmTH1* and VB1 show potential for improving both maize quality and stress tolerance.

## 2 Results

### 2.1 Identification of the *pldg1* Mutant

**FIGURE 1:**
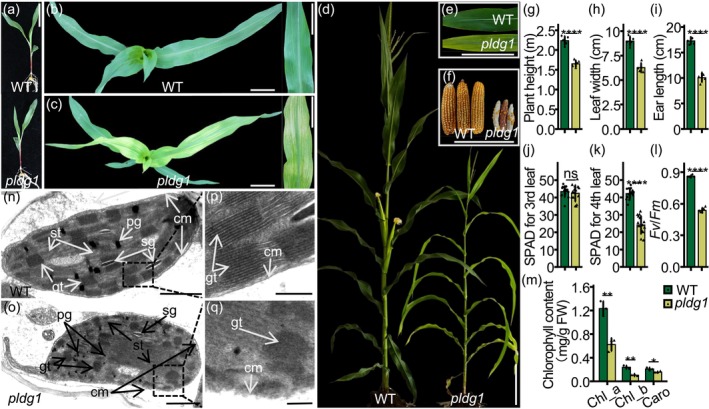
Phenotypes of *pldg1* in seedlings and adults. (a–c) Phenotypes of *pldg1* (*pale leaf and depauperate growth 1*) at the three‐leaf stage (a) and the five‐leaf stage (b, c). Scale bar = 5 cm. No phenotypic differences were observed between *pldg1* and WT (wild‐type) before the three‐leaf stage. (d–i) Phenotypes of *pldg1* at the adult stage. Scale bar = 20 cm. (j, k) Relative chlorophyll content (SPAD value) in the third (j) and fourth (k) leaves, respectively. (l) Chlorophyll fluorescence *F*_v_/*F*_m_ in the fourth leaves. (m) Chlorophyll content in the fourth leaves. Caro, carotenoid; Chl_a, chlorophyll a; Chl_b, chlorophyll b; FW, fresh weight. (n–q) Transmission electron micrographs of chloroplasts from fourth leaves of WT and *pldg1*. Scale bars: 1 μm (n, o); 0.25 μm (p, q). cm, Chloroplast membranegt, Granum‐thylakoid; pg., Plastoglobule; sg, Starch grain; st, Stroma‐thylakoid. Values are presented as means ± standard deviation (SD). *n* = 5 in (g–i, l), *n* = 15 in (j, k) and *n* = 3 in (m). ns, not significant. **, ***, ****: *p* < 0.01, Student's *t*‐test.

In an F_2_ population derived from a cross between maize inbred lines M66 and dg166, we identified a natural mutant line exhibiting chlorosis beginning at the fourth leaf stage. At maturity, mutant plants showed reduced plant height, narrower leaves and shorter ears compared with the wild type (WT) (Figure 1a–i). Based on these characteristics, the mutant was designated as *pale leaf and depauperate growth 1* (*pldg1*). To elucidate the physiological basis of the phenotype, we quantified chlorophyll levels and analysed chloroplast structure in seedling leaves. Soil plant analysis development (SPAD) measurements revealed no difference in relative chlorophyll content between WT and *pldg1* in the third leaf. However, a sharp decline was observed in the fourth leaf of *pldg1* (Figure 1j,k). Additionally, pigment analysis showed significant reductions in chlorophyll a, chlorophyll b and carotenoids, accompanied by a decrease in the maximum quantum yield of PSII (*F*_v_/*F*_m_) in the fourth leaf of *pldg1* (Figure 1l,m). Transmission electron microscopy revealed severe chloroplast abnormalities in *pldg1* leaves. Mutant chloroplasts were irregularly oval and dilated, with loosely folded granum‐thylakoids, indistinct stroma‐thylakoids and partially degraded chloroplast membranes (Figure 1n–q). These observations indicate that *pldg1* chloroplasts deteriorate from the fourth leaf stage, resulting in decreased chlorophyll content, reduced photosynthesis and chlorotic symptoms.

### 2.2 *ZmTH1* Is Essential for Normal Plant Growth

**FIGURE 2:**
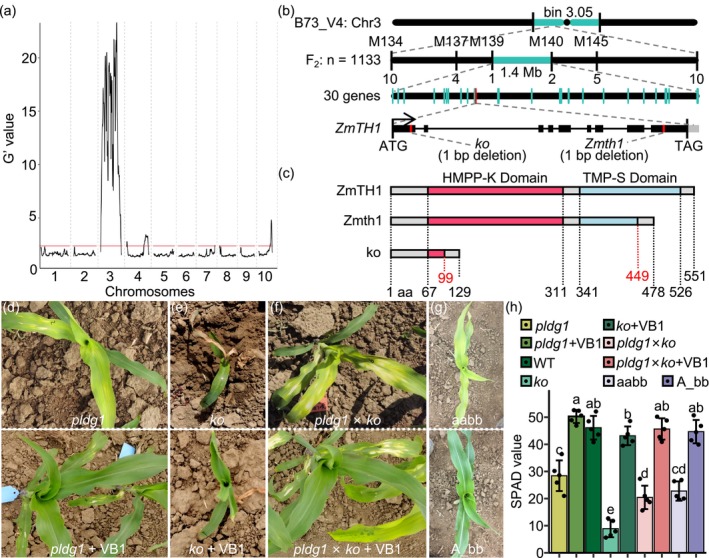
The VB1 synthetic gene *ZmTH1* causes *pldg1* symptoms. (a) BSA identified the genetic interval on chromosome 3. (b) Map‐based cloning revealed the gene *ZmTH1*. *Zmth1* exhibited a 1‐bp deletion in the ninth exon in *pldg1*, while the *ZmTH1* knockout line (*ko*) contained another 1‐bp deletion in the first exon. (c) Protein primary structure of *ZmTH1*, *Zmth1* and ko, red font indicates the mutation site. aa, Amino acid; HMPP‐K, Phosphomethylpyrimidine kinase; TMP‐S, Thiamine monophosphate synthase. (d–g) *pldg1*, *ko*, *pldg1* × *ko*, aabb and A_bb phenotype before and after VB1 supplementation. (h) SPAD value of different materials before and after VB1 supplementation. Values are presented as means ± standard deviation (SD). *n* = 5, different letters indicate significant differences (*p* < 0.01, One‐way ANOVA, LSD.test).

**FIGURE 3:**
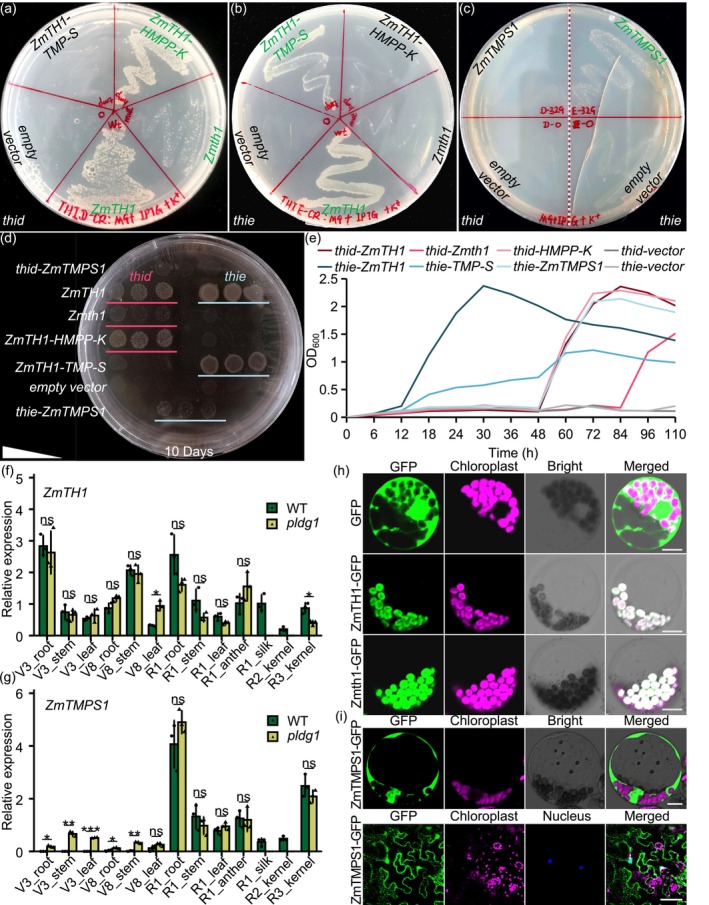
Functional verification of *ZmTH1* and *ZmTMPS1*. (a–c) Growth patterns of two *E.coli* deficient strains *thid* and *thie* heterologously expressing *ZmTH1*, *ZmTH1‐HMPP‐K*, *ZmTH1‐TMP‐S*, *Zmth1*, and *ZmTMPS1* sequences, respectively. Green font indicates viable strain growth. In (b), mutation of the TMP‐S domain in *Zmth1* impairs VB1 synthesis, consequently inhibiting *thie* growth. (d, e) Differential growth efficiencies among transgenic strains. Triangle indicates a concentration gradient from high to low in three replicates. *ZmTH1‐HMPP‐K* and *ZmTH1‐TMP‐S* represent HMPP‐K domain coding sequence and TMP‐S domain coding sequence of *ZmTH1*, respectively. (f, g) Relative expression levels of *ZmTH1* and *ZmTMPS1* in *pldg1* and WT across different tissues. R1, reproductive 1V3, vegetative 3. Values are presented as means ± standard deviation (SD). *n* = 3, ns, not significant. * *p* < 0.05. **, *** *p* < 0.01, Student's *t*‐test. Due to developmental defects in *pldg1*, silk and kernel were absent during R1 and R2 stages. (h) Subcellular localization of *ZmTH1* and *Zmth1* in maize leaf protoplast. Scale bar = 5 μm. (i) Subcellular localization of ZmTMPS1 in maize leaf protoplast (up) and tobacco leaf (down). Scale bars: 10 μm (up) and 50 μm (down).

To determine the genetic basis of *pldg1*, we performed segregation analysis in the F_2_ segregation. The ratio of WT and *pldg1* plants fits a 7:1 distribution (895:105, *χ* ^2^ = 3.66, *p* > 0.05), indicating that the mutations originated from a heterozygous recessive mutation in one parent. This finding was corroborated by the 1:1 ratio (263:225, *χ* ^2^ = 2.96, *p* > 0.05) observed in a BC_1_F_1_ population derived from crossing *pldg1* with wild‐type M66. To identify the target gene, bulk segregation analysis (BSA) was combined with map‐based cloning using the F_2_ population. BSA was conducted using two DNA pools of *pldg1* and WT, each containing 50 individuals, and a broad interval was identified on chromosome 3 (Figure 2a). To validate the interval and facilitate map‐based cloning, Insertion/Deletion (InDel) markers were designed based on variant call format (VCF) files from BSA. The interval was initially narrowed to bin3.05 containing the centromere using thousands of samples. Subsequently, genotyping of 1133 samples refined the interval to a 1.4 Mb region between markers M139 and M140, encompassing 30 annotated genes (Table S1). When further interval reduction proved unfeasible, coding sequence variations within these 30 genes were examined by analysing binary alignment/map (BAM) files from BSA. Only *Zm00001d041829* displayed a 1‐bp deletion in the ninth exon of *pldg1*, confirmed through sequencing (Figure 2b; Figure S1a,b). Given its orthology to *Arabidopsis THIAMINE REQUIRING 1* (*AtTH1*, *AT1G22940*), the maize gene was designated as *ZmTH1* and the mutant allele as *Zmth1*. *ZmTH1* functions as a bifunctional enzyme in VB1 biosynthesis, specifically catalysing HMP‐PP and TMP synthesis, containing HMPP‐K (Pfam number: PF08543) and TMP‐S (PF02581) domains. The 1‐bp deletion in *Zmth1* induced a frameshift mutation, producing a truncated protein lacking the TMP‐S domain, thereby disrupting VB1 synthesis. VB1 supplementation rescued the *pldg1* phenotype (Figures 2c and 3b; Figure S2). Thus, *Zm00001d041829* was identified as the candidate gene.

To further validate the authenticity of *ZmTH1*, we performed gene knockout experiments, allelic tests, genetic complementation tests. The knockout of *ZmTH1* in the B104 inbred line background targeted the first exon, resulting in a positive event with a 1‐bp deletion, which generated a truncated protein lacking both HMPP‐K and TMP‐S domains (Figure 2b,c). The *ZmTH1* knockout (*ko*) line exhibited albinism from the fourth leaf and subsequently wilted to death, a condition fully reversed by VB1 supplementation (Figure 2e). An allelic test conducted by crossing *pldg1* with *ko* produced F_1_ progeny displaying chlorosis symptoms, indicating that *pldg1* and *ko* were allelic, though different mutation sites caused distinct symptoms (detailed in subsequent sections). The lethality of *ko* resulted in F_1_ progeny resembling *pldg1*, which responded to VB1 supplementation (Figure 2f). Additionally, we performed a complementation test by crossing the *ZmTH1*‐overexpressed B104 line (designated AaBB, where Aa represents overexpression and BB represents wild‐type *ZmTH1*) with *pldg1* (aabb, where aa indicates non‐overexpression and bb represents mutant *Zmth1*). A transgenic marker and an allele‐specific PCR (ASP) marker, designed based on the 1‐bp deletion in *Zmth1*, distinguished between A_bb and aabb individuals in the F_2_ population (Figure S1c; Table S2). While aabb exhibited chlorosis, A_bb appeared normal, demonstrating that restoring *ZmTH1* in *pldg1* alleviated its symptoms (Figure 2g). SPAD values decreased in *pldg1*, *ko*, *pldg1* × *ko* and aabb, whereas VB1 supplementation or *ZmTH1* complementation restored these values (Figure 2h). These genetic analyses and VB1 supplementation experiments confirm *ZmTH1* as the target gene.

### 2.3 *ZmTH1* and *ZmTMPS1* Synergistically Function in VB1 Biosynthesis

To elucidate *ZmTH1* functions, we initially conducted phylogenetic analysis. Both HMPP‐K and TMP‐S domains demonstrated high conservation in plants, whereas in bacteria these domains typically existed separately, with *thiD* and *thiE* encoding HMPP‐K and TMP‐S in *E. coli*, respectively (Figure S3). Furthermore, *Zm00001d035329*, the paralog of *ZmTH1*, encodes exclusively a TMP‐S domain and maintains 94.6% identity within the TMP‐S domain of *ZmTH1* (Figures S3 and S4); thus, we designated it *ZmTMPS1*. The presence of *ZmTMPS1* potentially explains the differential phenotypes between *pldg1* and *ko*: in *pldg1*, *ZmTMPS1* potentially compensates for the lost TMP‐S function of *ZmTH1*, maintaining minimal VB1 synthesis and resulting in malnutrition without lethality. Conversely, the disruption of both HMPP‐K and TMP‐S domains in *ko* leads to lethality due to the complete loss of VB1 synthesis capability.

To test the hypothesis, we utilised CRISPR to knock out *thiD* and *thiE* in *E. coli* strain MG1655, generating two defective strains *thid* and *thie* (Figure S5). Subsequently, we heterologously expressed *ZmTH1*, *Zmth1* and *ZmTMPS1* in these strains to evaluate their functions. The results (Figure 3a–c) demonstrated that both *thid* and *thie* expressing *ZmTH1* exhibited growth on M9 medium, indicating that *ZmTH1* contains functional HMPP‐K and TMP‐S domains. In contrast, the expression of *Zmth1* restored growth only in the *thid* mutant, demonstrating that the mutant protein retains HMPP‐K but lacks TMP‐S activity. The HMPP‐K domain sequence of *ZmTH1* (*ZmTH1‐HMPP‐K*) restored *thid* growth, while the TMP‐S domain (*ZmTH1‐TMP‐S*) restored *thie*, confirming the independent functionality of the HMPP‐K and TMP‐S domains. Expression of *ZmTMPS1* restored growth in *thie* but not in *thid*, demonstrating that *ZmTMPS1* encodes TMP‐S activity exclusively. VB1 supplementation restored growth in all transgenic strains (Figure S6). Growth efficiency varied among transgenic strains (Figure 3d,e): for *thid*, the order was *ZmTH1*≈*ZmTH1‐HMPP‐K* > *Zmth1*, indicating that while *Zmth1* retains HMPP‐K function, the mutation reduces its activity. For *thie*, the order was *ZmTH1* > *ZmTH1‐TMP‐S* > *ZmTMPS1*, demonstrating that *ZmTMPS1* exhibits lower TMP‐S activity compared to *ZmTH1*. These results indicate that the HMPP‐K and TMP‐S domains of *ZmTH1* function independently; the HMPP‐K domain retains activity in *Zmth1*, whereas the TMP‐S domain is inactivated by the mutation. *ZmTMPS1* encodes a functional but less efficient TMP‐S enzyme. This explains the phenotypic difference between the two mutants: *pldg1* is chlorotic but viable because *ZmTMPS1* partially compensates for the loss of TMP‐S function, whereas the *ko* is lethal due to the disruption of both domains, which completely abolishes VB1 synthesis. These results systematically demonstrated the functions of the two domains in *ZmTH1* and its paralog *ZmTMPS1* in VB1 biosynthesis.

To elucidate *ZmTH1* and *ZmTMPS1* functions further, we analysed their temporal and spatial expression patterns using real‐time quantitative reverse transcription PCR (qRT‐PCR) across multiple tissues in the F_2_ population. The tissues examined included roots, stem apexes and leaves at V3 (vegetative 3), V8, and R1 (reproductive 1) stages, anthers and silks at the R1 stage, and kernels at R2 and R3 stages. *ZmTH1* expression was detected in all tested tissues with no significant differences between *pldg1* and WT (Figure 3f). Conversely, *ZmTMPS1* showed minimal expression in root, stem and leaf tissues of WT at V3 and V8 stages but demonstrated significant expression in *pldg1* (Figure 3g). After the R1 stage, *ZmTMPS1* expression was observed in all tissues of both WT and *pldg1* without significant differences, displaying an expression pattern similar to *ZmTH1*. These findings suggest that *ZmTMPS1* predominantly expresses in later developmental stages in WT and undergoes feedback regulation by *ZmTH1*. When *ZmTH1* loses TMP‐S function, *ZmTMPS1* expression is induced at earlier stages.

Previous studies have indicated that OsTH1 and AtTH1 localise to chloroplasts, while an N‐terminus‐truncated OsTH1 variant resides in the cytosol (Ajjawi, Tsegaye, and Shintani 2007; Nie et al. 2022), suggesting that the N‐terminus of *ZmTH1* might contain a chloroplast‐targeting signal. To investigate the localization of *ZmTH1* and *ZmTMPS1*, subcellular localization studies were performed. Transformation of maize leaf protoplasts with *Ubi::ZmTH1‐GFP* or *Ubi::Zmth1‐GFP* plasmid demonstrated that both *ZmTH1* and *Zmth1* localised to chloroplasts (Figure 3h), indicating that the 1‐bp deletion in *Zmth1* does not affect its localization. Notably, transformation of maize leaf protoplasts with *Ubi::ZmTMPS1‐GFP* and transient expression of *35S::ZmTMPS1‐GFP* in tobacco leaves revealed that *ZmTMPS1* localised to the cytosol and nucleus (Figure 3i). This subcellular distribution suggests that *ZmTMPS1* contributes to TMP synthesis outside the chloroplast, thereby expanding the known compartmentalization of VB1 biosynthesis. Collectively, these results indicate that *ZmTH1* and *ZmTMPS1* function in VB1 biosynthesis synergistically.

### 2.4 *ZmTH1* Globally Regulates TMP, VB1, TDP Levels and TDP‐Dependent Enzyme Activity

**FIGURE 4:**
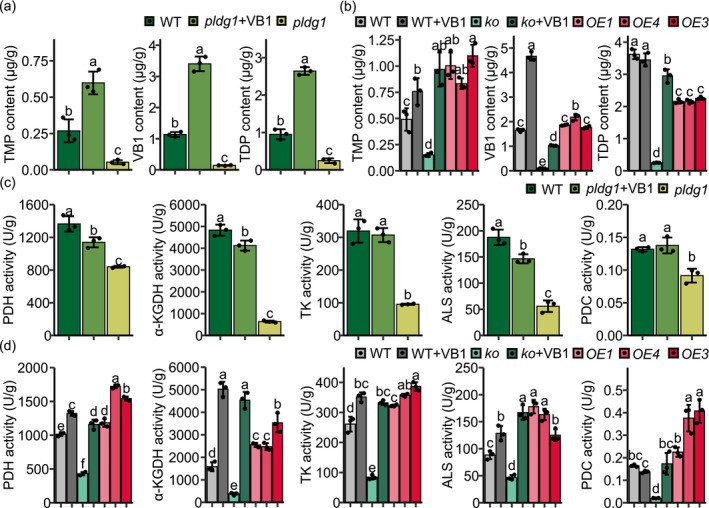
Changes in TMP, VB1 and TDP levels and TDP‐dependent enzyme activity. (a, b) TMP, VB1, and TDP levels in *pldg1* before and after VB1 supplementation (a) and in VB1 application and transgenic lines (b). (c, d) TDP‐dependent enzyme activity in *pldg1* before and after VB1 supplementation (c) and in VB1 application and transgenic lines (d). B104 serves as the wild‐type control in (b, d). ALS, acetolactate synthase; *ko*, *ZmTH1* knockout line; *OE*, *ZmTH1* overexpression line; PDC, pyruvate decarboxylase; PDH, pyruvate dehydrogenase; TDP, thiamin diphosphate; TK, transketolase; TMP, thiamin monophosphate; α‐KGDH, α‐ketoglutarate dehydrogenase. Values are presented as means ± standard deviation (SD). *n* = 3, different letters indicate significant differences (*p* < 0.01, One‐way ANOVA, LSD.test).

To directly examine the alterations in VB1 and its derivatives caused by *ZmTH1* mutations, mass spectrometry analysis was conducted. In *pldg1* and *ko*, the levels of TMP, VB1 and TDP decreased, while VB1 supplementation significantly restored their accumulation (Figure 4a,b). These results indicate that *ZmTH1* mutations impede TMP synthesis, subsequently affecting VB1 and TDP synthesis. To assess the effects of VB1 application and *ZmTH1* overexpression (*OE*) in normal plants, measurements were conducted for both treatments (Figure 4b). VB1 application increased TMP and VB1 levels without affecting TDP levels. Conversely, *OE* enhanced TMP levels and slightly improved VB1 levels but decreased TDP levels. These findings suggest that appropriate VB1 application can enhance VB1 and its derivatives in maize, while *ZmTH1* overexpression primarily increases TMP levels. TDP biosynthesis is regulated by both circadian rhythm and riboswitch, where excess TDP triggers feedback via the riboswitch in *THIC*, reducing *THIC* expression and TDP synthesis (Wachter et al. 2007; Bocobza et al. 2013; Noordally et al. 2020). Furthermore, TH2 exhibits TDP phosphatase activity, capable of dephosphorylating TDP to TMP. This connects TMP, VB1 and TDP in a cycle through TH2 and TPK to regulate TDP homeostasis (Hsieh et al. 2022). Our data are consistent with this regulatory cycle, as TMP and TDP were still detectable in *ko*, presumably derived from endosperm‐supplied VB1 through THIC, TH2, TPK activities.

Since TDP functions as a cofactor for various enzymes in multiple pathways, the activity of TDP‐dependent enzymes, including PDH, α‐KGDH, TK, ALS and PDC, was measured. These enzyme activities were diminished in *pldg1* and *ko*, while VB1 supplementation markedly restored them (Figure 4c,d), indicating that TDP reduction correspondingly decreases TDP‐dependent enzymes. Furthermore, both *OE* and VB1 application significantly enhanced the activities of TDP‐dependent enzymes (Figure 4d). The observed decrease in TDP levels in *OE* may reflect an adaptation to maintain TDP homeostasis, as the enhanced TDP‐dependent enzyme activity suggests a potential shift toward bound TDP utilisation. Together, these results showed that *ZmTH1* overexpression and VB1 application positively impact VB1 levels and the activity of TDP‐dependent enzymes. The *Zmth1* mutation directly reduces TMP synthesis, leading to decreased contents of VB1 and TDP, and subsequently decreases the activity of TDP‐dependent enzymes in *pldg1*.

### 2.5 *ZmTH1* Plays Important Roles in TDP‐Dependent Metabolic Pathways

**FIGURE 5:**
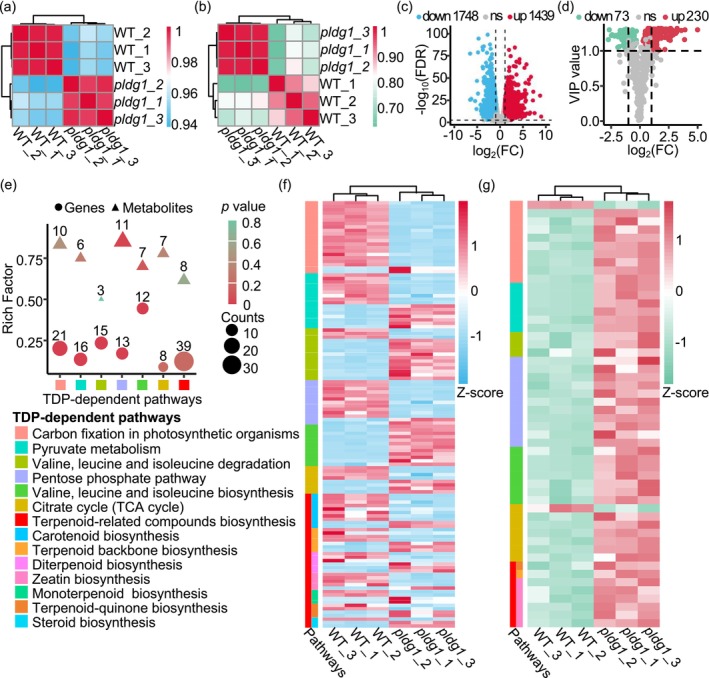
RNA‐seq and metabolomics revealed the disruption in TDP‐dependent pathways. (a, b) Sample correlation analyses of RNA‐seq (a) and metabolomics (b). (c, d) Volcano plots of RNA‐seq results (c) and metabolomic results (d). FC, fold change; FDR, false discovery rate; ns, not significant; VIP, variable importance in projection. (e) Differentially expressed genes/metabolites (DEGs/DEMs) in pathways involving TDP‐dependent enzymes. The rich factor is defined as the ratio of DEMs to the total metabolites annotated in that pathway, with higher values indicating stronger pathway enrichment. *p* value < 0.05 indicates significant enrichment of DEGs in the pathway. (f, g) Heatmaps of DEGs (f) and DEMs (g) in pathways involving TDP‐dependent enzymes.

To elucidate the alterations in TDP‐dependent metabolic pathways, RNA‐seq and primary metabolomics analyses were conducted using leaves from WT and *pldg1* in the F_2_ population. Correlation analysis revealed strong associations among replicates within identical samples, but weak associations between *pldg1* and WT, indicating robust and reliable results (Figure 5a,b). RNA‐seq identified 3187 differentially expressed genes (DEGs) (|log_2_FC| ≥ 1 and FDR < 0.05) in *pldg1*, comprising 1439 upregulated and 1748 downregulated genes. Kyoto Encyclopaedia of Genes and Genomes (KEGG) analysis of DEGs identified 45 significantly upregulated and 28 significantly downregulated pathways (*p* < 0.05) in *pldg1*, including BCAA synthesis and degradation, carbon fixation in photosynthetic organisms, pyruvate metabolism and PPP, which directly requires TDP‐dependent enzymes (Figure 5c and Figure S7). Metabolomics analysis detected 303 differentially expressed metabolites (DEMs) (VIP ≥ 1 and |log_2_FC| ≥ 1 or *p* < 0.05) in *pldg1*, with 73 decreased and 230 increased (Figure 5d). Although KEGG analysis did not identify significantly altered pathways due to the limited number of total metabolites in primary metabolomics, DEMs were observed in pathways related to TDP‐dependent enzymes (Figure S8). These findings indicate multiple disrupted TDP‐dependent pathways in *pldg1*.

We systematically analysed the changes in DEGs and DEMs involved in TDP‐dependent pathways (Figure 5e–g). Central carbon metabolism was strongly affected. The Calvin cycle (KEGG class: zma00710) had 21 DEGs and 10 DEMs (Figure S9), including five genes encoding ribulose‐1,5‐bisphosphate carboxylase/oxygenase (Rubisco) and phosphoribulokinase (PRK), and three metabolites directly involved in TDP‐dependent reactions like fructose‐6‐phosphate, erythrose‐4‐phosphate and sedoheptulose‐7‐phosphate. In pyruvate metabolism pathway (zma00620), 16 DEGs and 6 DEMs (Figure S10) were detected, including six genes involved in the conversion of pyruvate to phosphoenolpyruvate (PEP), oxaloacetate (OAA) and malate (Mal), and two metabolites Mal and PEP. The BCAA degradation pathway (zma00280) had 15 DEGs and 3 DEMs (Figure S11), including two BCAA members Leu and Ile. The PPP (zma00030) had 13 DEGs and 11 DEMs (Figure S12); the BCAA synthesis pathway (zma00290) had 12 DEGs and 7 DEMs (Figure S13). The TCA cycle pathway (zma00020) had 8 DEGs and 7 DEMs (Figure S14), including two metabolites α‐KGDH and citric acid; the terpenoid‐related compound synthesis pathways (including carotenoid biosynthesis (zma00906), terpenoid backbone biosynthesis (zma00900), diterpenoid biosynthesis (zma00904), zeatin biosynthesis (zma00908), monoterpenoid biosynthesis (zma00902), terpenoid‐quinone biosynthesis (zma00130) and steroid biosynthesis (zma00100)) had 39 DEGs and 8 DEMs (Figure S15). DEMs in these pathways were predominantly accumulated in *pldg1*, while DEGs varied widely (Figure 5f,g; Table S3). Additionally, we found 37 DEGs in the photosynthesis pathway (zma00195) and 15 DEGs in the oxidative phosphorylation pathway (zma00190), which included the formation of photosystems, ferredoxin, ATP synthase and NADH dehydrogenase, indicating the disruptions of fundamental energy supply systems for photosynthesis and respiration in *pldg1* (Figures S16 and S17). qRT‐PCR validation of 16 representative genes from these pathways confirmed the RNA‐seq trends (Figure S18; Table S4), confirming the reliability of RNA‐seq. These findings suggest that the dysfunction of *ZmTH1* profoundly disrupts TDP‐dependent pathways, leading to dysregulation in other pathways and ultimately resulting in *pldg1*symptoms.

### 2.6 *ZmTH1* Overexpression and Exogenous VB1 Application Enhance Cold and Drought Tolerance in Seedlings

Previous research has demonstrated that VB1 synthetic gene overexpression and VB1 application enhance tolerance to various biotic and abiotic stresses in multiple plant species. However, this phenomenon's occurrence in maize remains unexplored. The present study reveals that *ZmTH1* overexpression and VB1 application increased TDP‐dependent enzyme activity (Figure 4d), potentially enhancing the efficiency of relevant pathways, particularly those involving ALS, BCKDH and TK in BCAA metabolism and PPP (see Section 3). These metabolic enhancements may strengthen plant innate immunity.

**FIGURE 6:**
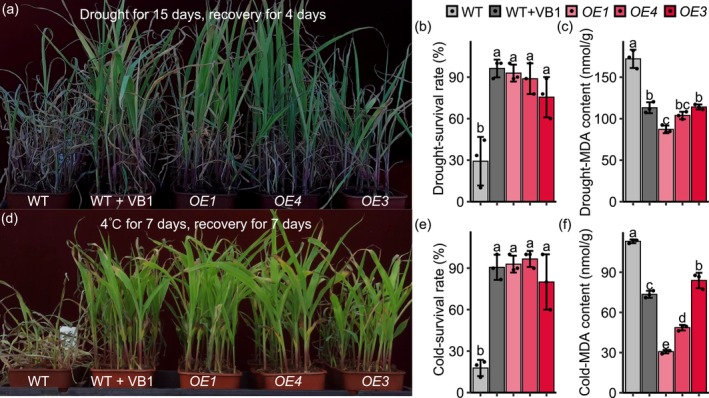
*ZmTH1* overexpression or VB1 application enhanced cold and drought tolerance. (a, d) Phenotypic responses of VB1‐treated or *ZmTH1* overexpression (*OE*) seedlings under cold and drought stress conditions. (b, c, e, f) Survival rates and MDA concentrations in VB1‐treated or *ZmTH1* overexpression seedlings under cold and drought stress conditions. B104 serves as the wild‐type control. Values are presented as means ± standard deviation (SD). *n* = 3, different letters indicate statistically significant differences (*p* < 0.01, One‐way ANOVA, LSD.test).

To investigate whether *ZmTH1* overexpression or VB1 application enhances maize tolerance to abiotic stresses, multiple stress treatments (salt, alkali, cold, heat, drought, flood) were conducted on maize seedlings. The B104 inbred line for *OE* served as a wild‐type control. Expression levels of the three *OE* lines are presented in Figure S19. No significant differences were observed among WT, WT + VB1, *OE* plants under salt, alkali, heat or flooding stresses. In contrast, marked differences were detected under drought and cold stresses (Figure 6a,d). After rehydration following drought treatment or rewarming following cold treatment, the survival rates of WT + VB1 and *OE* reached ~90%, compared with only ~30% in WT controls (Figure 6b,e). Correspondingly, the MDA content, which reflects overall oxidative stress level, was significantly lower in WT + VB1 and *OE* (Figure 6c,f), indicating enhanced antioxidant capacities. These results manifest that *ZmTH1* overexpression and exogenous VB1 application can enhance maize tolerance to drought and cold stresses in maize, providing a potential gene target for molecular breeding and highlighting the value of VB1 as a foliar fertiliser.

## 3 Discussion

In this study, we identified a critical VB1 biosynthetic gene *ZmTH1* and its paralog *ZmTMPS1*. The broad mapping interval for *ZmTH1* observed in BSA analysis likely reflects its proximity to the centromere, where reduced recombination frequency limits mapping resolution (Naish et al. 2021; Li and Xu 2022). The alteration in *ZmTH1* protein structure likely attenuates its HMPP‐K activity (Figure S20), as structural changes affect substrate binding and thereby decrease enzyme activity (Garcia et al. 2017; Lonhienne et al. 2018). The non‐lethal phenotype of *pldg1* can be explained by functional compensation from *ZmTMPS1*, which provides TMP‐S activity. This scenario parallels VB1 synthetic gene mutants in soybean (*pgl1*) and maize (*blk1‐R*), both of which are partially rescued by their paralogs (Woodward et al. 2010; Feng et al. 2019). Mutations in Os*TH1* and *AtTH1* led to seedling lethality (Ajjawi, Tsegaye, and Shintani 2007; Nie et al. 2022), and disruption of both HMPP‐K and TMP‐S domains in *ko* also proved lethal (Figure 2b–e). These findings underscore the indispensable role of the conserved *ZmTH1* in maize VB1 synthesis and indicate that *ZmTMPS1* can partially complement *ZmTH1* by supplying TMP‐S activity.

VB1 synthetic proteins exhibit diverse subcellular locations. AtTHI1 protein targets both mitochondria and chloroplasts while performing DNA damage repair functions (Chabregas et al. 2001, 2003). Under stress conditions, THI1 in wheat and *Arabidopsis* relocates from chloroplasts to the plasma membrane (Li et al. 2016; Yang et al. 2023), indicating that multi‐localised THI1 participates in VB1 synthesis, DNA repair and stress responses. Additionally, AtTH2 protein localises to mitochondria, cytosol and nucleus, functioning as a TMP phosphatase and potentially as a TDP phosphatase (Mimura et al. 2016; Hsieh et al. 2017, 2022). Our subcellular localization results indicate that, in addition to TMP synthesis by *ZmTH1* in chloroplasts, *ZmTMPS1* can catalyse TMP in the cytosol and nucleus. In *Arabidopsis*, TPK protein localises to the cytosol (Ajjawi, Rodriguez Milla, et al. 2007). In maize, three annotated TPK genes (*Zm00001d037916*, *Zm00001d040798*, *Zm00001d042241*) need to be clarified. Given that TMPS1 and TH2 proteins localise to multiple organelles, we hypothesize that TPKs may also exhibit multi‐localization, enabling TDP synthesis in different organelles. Experimental validation of this hypothesis will be important to fully elucidate the spatial organisation of VB1 metabolism in maize.

TDP‐dependent metabolisms play a fundamental and essential role in plant biology. The Calvin cycle fixes CO_2_ and provides carbon resources for plants, with PRK and Rubisco being crucial for initiating CO_2_ fixation (Yu et al. 2020; Ludwig et al. 2024). Pyruvate functions as a central intermediate connecting carbohydrate, amino acid and fatty acid metabolisms and can interconvert with PEP, Mal and OAA to participate in various metabolic processes (Gray et al. 2014). BCAAs are closely related to pyruvate metabolism, the TCA cycle and oxidative phosphorylation, and they are involved in plant responses to abiotic stresses (Binder 2010; Angelovici et al. 2013; Galili et al. 2016). BCAA accumulation is frequently observed under abiotic stresses like drought, heat and light (Bowne et al. 2012; Obata and Fernie 2012; Galili et al. 2016). Moreover, the overexpression of BCAA metabolic genes or BCAA application enhances drought or salt tolerance in rice (Shim et al. 2023; Sun et al. 2024). The PPP is a primary defence against oxidants in plants, and it is tightly linked to glycolysis and the syntheses of fatty acid, sterol, amino acid, nucleotide (Kruger and von Schaewen 2003; Stincone et al. 2015). The NADPH produced by PPP acts as a reducing equivalent for glutathione reductase, peroxidases and thioredoxins, thereby supporting reactive oxygen species (ROS) scavenging under stress (Valderrama et al. 2006; Patra and Hay 2014; Stincone et al. 2015). Mutations in the *Arabidopsis* NADH kinase gene *nadk3* lead to decreased NADPH levels, increasing sensitivity to oxidative, salt and osmotic stresses (Chai et al. 2006). Conversely, overexpression of a NADPH upstream gene *cP2* enhances cytosolic NADPH levels, improving tobacco's resistance to *Phytophthora* and drought (Scharte et al. 2009). The TCA cycle is the final aerobic oxidation pathway, producing NADH and FADH_2_, which fuel oxidative phosphorylation to generate large amounts of ATP, the primary energy for basal metabolisms. Intermediates from the TCA cycle also contribute carbon skeletons to fatty acid, amino acid and nucleotide biosynthesis (Sweetlove et al. 2010; Araujo et al. 2012). Isoprenoids, produced by the MEP pathway, constitute the largest class of organic compounds in nature. Many plant hormones like cytokinins, gibberellins, abscisic acid, strigolactones, brassinosteroids require isoprenoids (Zhao et al. 2013; Pu et al. 2021). Therefore, disruptions in these TDP‐dependent pathways caused by *Zmth1* trigger a series of reactions, leading to multiple metabolic abnormalities in *pldg1*. Improving the efficiency of these pathways contributes to plant growth.

The isoprenoid precursor 1‐deoxy‐D‐xylulose‐5‐phosphate (DXP), synthesised through the MEP pathway in plastids, is essential for the biosynthesis of chlorophylls and carotenoids (Arigoni et al. 1997; Eisenreich et al. 2001). A mutation in the DXS gene produced an albino phenotype that was restored by supplementing 1‐deoxy‐D‐xylulose (DX), while the chlorosis induced by TK gene overexpression was remediated by DXP (Estevez et al. 2000; Khozaei et al. 2015). Additionally, a mutation in the DXP reductoisomerase gene resulted in leaf bleaching, and flux from the cytosolic mevalonate pathway proved insufficient to ameliorate the defects (Xing et al. 2010). These findings demonstrate the critical role of isoprenoids from the MEP pathway in photosynthetic pigments. Consequently, we treated *ko* with exogenous DX after germination. Regular supplementation of DX to both roots and leaves partially alleviated the albino symptom and delayed lethality (Figure S21), and the DX treatment significantly elevated the levels of chlorophyll a, chlorophyll b and carotenoids in *ko*. This indicates that the lethal albino phenotype of *ko* directly results from inhibition of the MEP pathway mediated by DXS.

**FIGURE 7:**
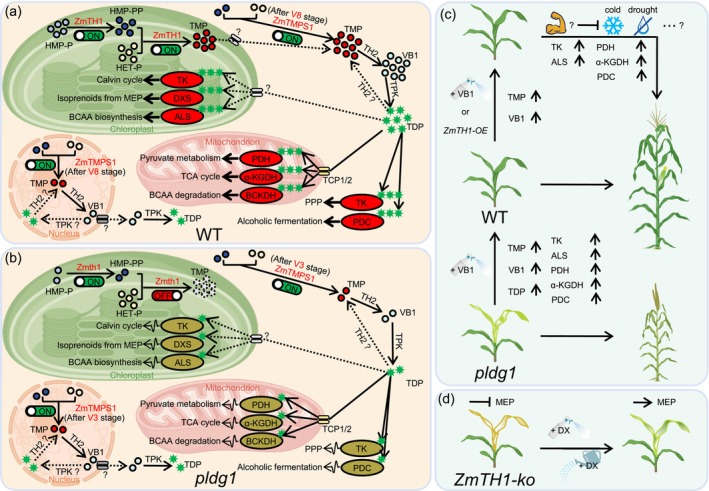
Models of *ZmTH1* and *ZmTMPS1* roles in maize VB1 synthesis and growth. (a) In WT plants, *ZmTH1* and ZmTMPS1 contribute to TMP synthesis. *ZmTH1* functions continuously in the chloroplast, whereas ZmTMPS1 functions in the cytosol and nucleus after the V8 stage. VB1s, synthesised by TH2, are converted to TDPs by TPK, which then bind to TDP‐dependent enzymes in various organelles, ensuring proper pathway function and promoting healthy growth. (b) In *pldg1*, *Zmth1* synthesises limited HMP‐PPs and cannot produce TMP. ZmTMPS1 initiates minimal TMP synthesis from an earlier stage, resulting in restricted VB1s and TDPs production. The reduced TDP binding to TDP‐dependent enzymes leads to decreased enzyme activity, disrupted pathways and impaired growth. (c) *pldg1* displays seedling chlorosis and stunted adult growth. VB1 supplementation restores TMP, VB1 and TDP levels, enhancing TDP‐dependent enzyme activity and normalising *pldg1* growth. In WT, VB1 application or *ZmTH1*‐OE increases TMP and VB1 levels and TDP‐dependent enzyme activity, potentially enhancing pathway efficiency and plant immunity, thereby improving seedling tolerance to cold and drought, and possibly other stresses. (d) Exogenous DX alleviates the lethal albino symptom in *ZmTH1*‐*ko* line by restoring the MEP pathway. ALS, acetolactate synthase; BCAA, branched chain amino acids; BCKDH, branched chain keto‐acid dehydrogenase; DXS, 1‐deoxy‐D‐xylulose‐5‐phosphate synthase; HET‐P, hydroxyethylthiazole phosphate; HMP‐P, hydroxymethylpyrimidine phosphate; HMP‐PP, hydroxymethylpyrimidine pyrophosphate; *ko*, *Knockout*; MEP, methylerythritol phosphate; *OE*, *Overexpression*; PDC, pyruvate decarboxylase; PDH, pyruvate dehydrogenase; PPP, pentose phosphate pathway; TCA, tricarboxylic acid; TDP, thiamin diphosphate; TK, transketolase; TMP, thiamine monophosphate; V8, vegetative 8 stage; VB1, vitamin B1; α‐KGDH, α‐ketoglutarate dehydrogenase. Dashed lines and question marks indicate interactions that are hypothetical and have not yet been experimentally confirmed.

Based on our experimental findings and analysis, we propose models elucidating the roles of *ZmTH1* and *ZmTMPS1* in maize VB1 biosynthesis and development. In wild‐type plants, *ZmTH1* synthesises HMP‐PPs and TMPs in the chloroplast, which are subsequently transported to the cytosol, where TH2 converts TMPs to VB1. Furthermore, *ZmTMPS1* exhibits limited TMP‐S activity and produces minimal TMPs post‐V8 stage in the cytosol and nucleus, where TH2 converts these TMPs to VB1. TPK then converts all VB1 molecules to active TDPs in the cytosol. TH2 potentially exhibits TDP phosphatase activity for TMP regeneration. The subcellular distribution of TPK‐encoding proteins in maize remains undetermined. Active TDPs are distributed to various organelles, where they associate with TDP‐dependent enzymes, facilitating relevant metabolic pathways and supporting maize development (Figure 7a). In contrast, the *pldg1* mutation results in diminished HMPP‐K activity and eliminated TMP‐S activity of *Zmth1*, restricting HMP‐PPs synthesis and inhibiting TMP formation. *ZmTMPS1* initiates TMP synthesis earlier to compensate for *Zmth1* deficiency. Reduced total VB1 levels result in decreased TDP availability and TDP‐enzyme binding, diminishing enzyme activity and disrupting associated pathways, manifesting as seedling chlorosis and stunted growth (Figure 7b,c). VB1 supplementation elevates TMP, VB1 and TDP levels, thereby enhancing TDP‐dependent enzyme activity and restoring *pldg1* growth. *ZmTH1* overexpression or exogenous VB1 administration further increases TMP and VB1 levels and TDP‐dependent enzyme activity in wild‐type material. This enhancement of TDP‐dependent pathways may improve plant immunity and stress tolerance, particularly to cold and drought conditions (Figure 7c). The lethal albino phenotype in the *ZmTH1* knockout line results directly from MEP pathway inhibition, which exogenous DX application ameliorates (Figure 7d).

## 4 Experimental Procedures

### 4.1 Plant Materials

For *ZmTH1* cloning, maize inbred line M66 was crossed with dg166. The F_1_ progeny underwent self‐pollination to generate an F_2_ population, from which *pldg1* mutants were identified. To confirm segregation ratios, a BC_1_F_1_ population was generated by crossing *pldg1* with wild‐type M66, using *pldg1* as the recurrent parent. For allelic testing, the *ZmTH1* knockout line (*ko*) was crossed with *pldg1*. For genetic complementation, the *ZmTH1* overexpression line (AaBB) was crossed with *pldg1* (aabb), where Aa denotes overexpression, aa denotes non‐overexpression, BB represents wild‐type *ZmTH1* and bb represents mutant *Zmth1*. F_2_ individuals with genotypes A_bb (complementation lines) and aabb were identified using overexpression markers and ASP markers (Table S2).

The B104 inbred line served as the source for *ko* and *OE*. The Z31 inbred line provided leaf tissue for protoplast isolation. Field cultivation employed 10 m row lengths, with alternating narrow (40 cm) and wide (70 cm) rows, maintaining plant spacing of 30–40 cm after thinning.

### 4.2 VB1 Application

For *pldg1* mutants and *ko* lines, 5–10 mL of 0.1 g/L thiamine hydrochloride (Aladdin, Cat# T104106) solution was applied per plant as a foliar spray, beginning at the five‐leaf stage and repeated every 10 days until the R1 stage. In the wild‐type B104, thiamine hydrochloride was applied with gradient concentrations (0.1 g/L at the five‐leaf stage, 0.2 g/L at the V8 stage, 0.4 g/L at the R1 stage) to evaluate its potential effects on yield and quality traits. No VB1 was applied to *OE* lines.

### 4.3 Chlorophyll Content Measurement

For SPAD measurements, a Konica Minolta SPAD‐502 Plus instrument was utilised to analyse the leaves. Chlorophyll content determination employed ethanol solvent following the method of Lichtenthaler and Wellburn (1983). Briefly, leaf samples were pulverised in liquid nitrogen, and 0.1 g was extracted with 10 mL of 95% ethanol, followed by dark incubation for 30 min. Following centrifugation, supernatant absorbance was measured at 665 nm, 649 nm and 470 nm. The concentrations of chlorophyll a (Ca), chlorophyll b (Cb) and carotenoids (Cc) were determined using the following equations:

$$\mathrm{Ca}=13.95\times {\mathrm{OD}}_{665}–6.88\times {\mathrm{OD}}_{649}$$

$$\mathrm{Cb}=24.96\times {\mathrm{OD}}_{649}–7.32\times {\mathrm{OD}}_{665}$$

$$\mathrm{Cc}=\left(1000\times {\mathrm{OD}}_{470}–2.05\times \mathrm{Ca}—114.8\times \mathrm{Cb}\right)÷245$$

$$\begin{array}{c} \text{Content}\;\left(\mathrm{mg}/g\right)=\text{Concentration}\times \text{Volume}\\ \;÷\left(\text{Fresh Weight}\times 1000\right) \end{array}$$

For *F*_v_/*F*_m_ measurements, leaf samples were excised, dark‐adapted for 30 min on ice and measured using a WALZ PAM2100 instrument.

### 4.4 Transmission Electron Microscopy

Leaf samples (4–9 mm^2^) from the fourth leaves of *pldg1* and WT in the F_2_ population underwent vacuum infiltration with glutaraldehyde and fixation at 25°C for 48 h. Subsequently, samples underwent rinsing, replacement, dehydration, embedding and sectioning as previously described (Huo et al. 2020). Images were acquired using a HITACHI H‐7500 transmission electron microscope.

### 4.5 BSA and Map‐Based Cloning

For BSA, high‐quality DNA was extracted from leaves of 50 WT and 50 *pldg1* individuals in the F_2_ population, and 30 M66 individuals. DNA was combined equimolarly within each pool and prepared for whole‐genome resequencing using the DNBSEQ‐T7 platform with 150 bp paired‐end reads (Annoroad). Sequencing depth was 50× for F_2_ and 30× for M66. Raw data underwent quality assessment using FastQC (v0.11.9) and filtering via Fastp (v0.23.2). Reads were aligned to the B73_V4 reference genome (ftp://ftp.ensemblgenomes.org/pub/release‐50/plants/fasta/zea_mays/dna/Zea.mays.B73_RefGen_v4.dna.toplevel.fa.gz) using BWA (v0.7.17), and BAM files were generated using Samtools (v1.17). VCF files for variant calling were generated using GATK (v4.2.0.0). SNP and InDel filtering were performed using the recommended parameters. BSA was conducted using the R (v4.1.3) packages QTLseqr (v0.7.5.2) and vcfR (v1.13.0). A sliding window size of 4e6 and 10 000 permutations were used. The interval was identified based on the *Gprime* value.

For map‐based cloning, InDels with |deltaSNP| > 0.7 on chromosome 3 were extracted from VCF files. BAM files were visualised by IGV (v2.12.3) to confirm these InDels, and primers were designed for map‐based cloning. Polymorphic markers were identified between parents M66 and *dg166*. These markers were utilised to genotype *pldg1* individuals in the F_2_ population, and recombinants were selected to further narrow the interval. IGV was employed to identify genes differing between WT and *pldg1*.

### 4.6 Phylogenetic and Bioinformatics Analysis

*ZmTH1* homologues in other plants were retrieved from the Ensembl database using its B73_V5 designation *Zm00001eb138710* (https://ensembl.gramene.org/). Homologues in other species were obtained from NCBI (https://www.ncbi.nlm.nih.gov/) using BLASTP with the ‘model organisms (landmark)’ database. A phylogenetic tree was constructed using MEGAX (v10.1.8) with the neighbour‐joining method and refined with EvolView (http://www.evolgenius.info/evolview/). Protein domain analysis was conducted using Pfam (http://pfam‐legacy.xfam.org/) and InterPro (https://www.ebi.ac.uk/interpro/). Protein structure prediction and visualisation were performed using Phyre2 (http://www.sbg.bio.ic.ac.uk/phyre2/) and SPDBV (v4.1.0). Sequence comparisons were executed using DNAMAN (v9.0.1) and EMBOSS Needle (https://www.ebi.ac.uk/jdispatcher/psa/emboss_needle).

### 4.7 Overexpression and Knockout of *ZmTH1*

For overexpression, the full‐length coding sequence of *ZmTH1* was amplified from B73 cDNA and cloned into the pCAMBIA23001 vector driven by the *Ubiquitin* (*Ubi*) promoter. For knockout, a CRISPR/Cas9 knockout target site was designed in the first exon of *ZmTH1* to generate the guide RNA, which was subsequently ligated into the pBUE411 vector. Recombinant plasmids were transformed into the *Agrobacterium tumefaciens* strain EHA105 to infect the immature embryo of B104, generating *ZmTH1‐OE* or *ZmTH1‐ko* seedlings. Primers are listed in Table S2. Expression levels of *ko* and *OE* lines are shown in Figure S19.

### 4.8 Knockout of *thiD* and *thiE* in *E. coli* Strain MG1655

To validate *ZmTH1*, *Zmth1* and *ZmTMPS1* functions, *thiD* and *thiE* were knocked out in *E. coli* following previously described methods (Huang et al. 2020). The procedure for knocking out *thiD* is briefly described: the *thiD* sequence was obtained from EcoCyc (https://ecocyc.org/). CRISPR/Cas9 target primers were designed using CHOPCHOP (http://chopchop.cbu.uib.no/), with amplification based on the P09002 plasmid (Genestar Bio) template. Primers for amplifying the left and right homology arms required for homologous recombination were designed using the MG1655 strain (ZOMANBIO) as the template. These PCR products were ligated to generate the recombinant P09002 plasmid containing the knockout target and homology arms. The P09001 plasmid (Genestar Bio), containing the CRISPR/Cas9 system and homologous recombination repair components, was transformed into the MG1655 strain. Subsequently, the recombinant P09002 was introduced into the MG1655 strain harbouring P09001 to achieve *thiD* knockout. An identical procedure was applied for *thiE*. Primers are listed in Table S2.

### 4.9 Heterologous Expressions of *ZmTH1* , *Zmth1* and *ZmTMPS1* in *E. coli*

*ZmTH1*, *Zmth1*, *ZmTMPS1*, HMPP‐K domain of *ZmTH1* (*ZmTH1‐HMPP‐K*), TMP‐S domain of *ZmTH1* (*ZmTH1‐TMP‐S*) coding sequences were amplified from B73 or *pldg1* cDNA and inserted into the pet28a vector, which was induced by 0.8 mM isopropyl β‐D‐1‐thiogalactopyranoside (IPTG). The recombinant plasmids were transformed into *E. coli* defective strains *thid* and *thie*, respectively. Equal volumes of bacterial cultures, grown to OD_600_ = 0.5, were placed onto M9 solid media with or without 0.2 mM VB1 and incubated at 37°C for 2–10 days. For growth efficiency analysis, cultures were grown to OD_600_ = 0.5 and serially diluted (1×, 1/20×, 1/400×) before placement on M9 solid media. Furthermore, 20 μL of undiluted culture was inoculated into 20 mL of M9 liquid media without VB1 and incubated at 37°C, with OD_600_ absorbance measured every 6 or 12 h to monitor bacterial density. Primers are listed in Table S2.

### 4.10 RNA Extraction and qRT‐PCR

To analyse the expression patterns of *ZmTH1* and *ZmTMPS1*, RNA was extracted from roots, stem apexes, leaves, silks, anthers and developing kernels of *pldg1* and WT individuals at V3, V8, R1, R2 and R3 stages. Samples from three plants were combined for each biological replicate to minimise F_2_ background effects. Total RNA was extracted using Trizol (Gibco) and reverse transcribed into cDNA using the RevertAid First Strand cDNA Synthesis Kit (Thermo). qRT‐PCR was performed using 2× RealStar Green Fast Mixture (GenStar) on a LightCycler 480 II (Roche). The maize GAPDH gene (*Zm00001d049641*) served as an internal control. Relative expression levels were calculated using the $2^{-\Delta \Delta C_{T}}$ method (Schmittgen and Livak 2008), with three biological replicates per treatment. Primers are listed in Table S2.

To validate the reliability of RNA‐seq, qRT‐PCR was performed on eight significantly upregulated and eight significantly downregulated genes in *pldg1*. These genes included key genes from significantly altered KEGG pathways and VB1 biosynthetic genes (Table S4).

### 4.11 Subcellular Localization

The coding sequences of *ZmTH1*, *Zmth1* and *ZmTMPS1* lacking stop codons were amplified from B73 or *pldg1* cDNA and cloned in‐frame with GFP into the pJIT163‐Ubi‐hGFP vector for maize protoplast or the pCAMBIA1300‐35S‐GFP vector for tobacco. These vectors were transiently expressed in maize leaf protoplasts or tobacco leaves. Protoplasts were incubated for 14 h and tobacco leaves for 48 h before imaging. GFP was excited at 488 nm and emission was collected at 500–550 nm for GFP and 600–700 nm for chlorophyll autofluorescence. Nuclear localization was verified using DAPI staining with 405 nm excitation and 450–500 nm emission. Primers are listed in Table S2.

### 4.12 Measurement of TMP, VB1 and TDP Content

Samples from the fifth leaves of WT, *pldg1*, *pldg1* + VB1, B104, B104 + VB1, *ko*, *ko* + VB1 and *OE1*, *OE3*, *OE4* materials were collected at the V3 stage and subsequently ground in liquid nitrogen. Each sample included three biological replicates. For WT, *pldg1* and *pldg1* + VB1, leaves from three plants were combined for each biological replicate to minimise F_2_ background effects. Deionised water used in this section was purified by the Milli‐Q IQ7000 (Merk, Germany) water purification system (resistivity ≥ 18.2 MΩ).

For VB1 content determination, approximately 100 mg samples underwent overnight extraction at 4°C in water containing 20 ng of [^2^H_3_]‐VB1 as an internal standard. The crude extracts underwent further purification using an Oasis WCX SPE cartridge (3 cc, 60 mg) (Waters, Milford, MA, USA), preconditioned sequentially with methanol (MeOH) and water. Subsequently, the cartridge was washed with 20% MeOH and MeOH, followed by elution with 2% FA in 90% MeOH. The eluates were reconstituted in 0.1% FA in 90% acetonitrile (ACN) and analysed using a liquid chromatography–tandem mass spectrometry (LC–MS/MS) system consisting of an ACQUITY UPLC (Waters, Milford, MA, USA) and Quattro Premier XE MS (Waters, Manchester, UK) equipped with an electrospray (ESI) source. Five microliters of each sample were injected onto a BEH HILIC column (2.1 mm × 100 mm, 1.7 μm). The UPLC method utilised mobile phase A, 20 mM ammonium acetate (CH_3_COONH_4_) in water (pH = 8.5) and B, ACN. The flow rate was maintained at 0.3 mL/min with the column temperature at 30°C. The gradient proceeded as follows: 0–10 min, 100% B to 50% B; 10–11 min, 50% B; 11–12 min, 50% B to 100% B; 12–14 min, 100% B. The ESI source parameters were: capillary voltage, 3.0 KV; sampling cone voltage, 30 V; extraction cone voltage, 3 V; source temperature, 120°C; desolvation temperature, 400°C; desolvation gas, 800 L/h; and cone gas, 50 L/h. VB1 detection employed positive multiple reaction monitoring (MRM) mode. The MRM transitions for quantification were: 265.1 > 122.1 for VB1, 268.1 > 125.1 for [^2^H_3_]‐VB1.

For TMP and TDP content analysis, including both free and protein‐bound forms, approximately 100 mg samples were extracted for 30 min at 74°C in 1 mL of 0.1 M HCl containing 40 ng of [^2^H_3_]‐TMP and 334 ng of [^2^H_3_]‐TDP according to a previous report (Verstraete et al. 2020). The samples were cooled on ice for 2 min and centrifuged at 14 000 *g* for 15 min at 4°C. The filtered crude extract (0.22 μm syringe filters) was pH‐adjusted to 7 and diluted with water to a final volume of 3 mL. Further purification involved loading samples onto connected C18 (Waters Sep‐Pak, 3 cc, 200 mg)–WAX (Waters Oasis, 3 cc, 60 mg) SPE cartridges, preconditioned with MeOH and water. The WAX cartridge, separated from the C18 cartridge, underwent water washing and final elution with 2% TFA in 90% MeOH to obtain TMP and TDP fractions. The eluates were reconstituted in 0.1% FA and analysed using the same LC–MS/MS system as VB1 analysis. Five microliters of each sample were injected onto an HSS T3 column (2.1 mm × 100 mm, 1.7 μm). The UPLC method employed mobile phase A: 2 mM *N,N*‐Dimethylhexylamine in water (pH = 8.5), B: ACN. The flow rate was maintained at 0.3 mL/min with the column at 30°C. The gradient proceeded: 0–2 min, 0% B; 2–12 min, 0% B–40 B; 12–13 min, 40% B to 0% B; 13–20 min, 0% B. The ESI source parameters matched those for VB1 analysis. The MRM transitions for quantification were: 345.1 > 122.1 for TMP, 348.1 > 125.1 for [^2^H_3_]‐TMP, 425.1 > 122.1 for TDP and 428.1 > 125.1 for [^2^H_3_]‐TDP.

### 4.13 Measurement of TDP‐Dependent Enzyme Activity

Samples from ‘Measurement of TMP, VB1, and TDP content’ were utilized for this analysis. Enzyme activities were quantified using the following commercial kits: PDH Activity Assay Kit (Solarbio), α‐Ketoglutarate Dehydrogenase (α‐KGDH) Activity Assay Kit (Solarbio), Transketolase ELISA Kit (Camilo), ALS Activity Assay Kit (Solarbio), PDC Activity Assay Kit (Solarbio).

### 4.14 RNA‐Seq

Total RNA was extracted from the fifth leaves of *pldg1* and WT in the F_2_ population at the V3 stage, where leaves from 25 plants were pooled for each biological replicate to minimise F_2_ background effects, with three biological replicates. For RNA‐seq, libraries were prepared and sequenced on the DNBSEQ‐T7 platform using paired‐end reads (BGI Tech). Clean data was aligned to the maize B73_v4 reference genome by HISAT2 (v2.2.1). Read counts for each gene were obtained by FeatureCounts in Rsubread (v2.8.2) and normalised to FPKM (fragments per kilobase million) and TPM (transcripts per million) in edgeR (v3.36.0), and further normalised to TMM (trimmed mean of M‐values) by perl scripts. Differential expression analysis was performed using the ‘run_DE_analysis.pl'script in Trinity (v2.11.0) with the edgeR method. DEGs were identified with the threshold of |log_2_FC| ≥ 1 and FDR < 0.05.

### 4.15 Metabolome Analysis

0.5 g of each biological replicate used in RNA‐seq was sent to Metware Biotech for primary metabolome analysis. Samples were ground under freeze‐dried vacuum conditions, extracted with 70% methanol and analysed by the UPLC–MS/MS (ultra‐performance LC–MS/MS) system. The UPLC was equipped with an Agilent SB‐C18 column (1.8 μm, 2.1 mm × 100 mm). The mobile phase consisted of pure water with 0.1% formic acid and acetonitrile with 0.1% formic acid. A gradient elution program was employed, with a flow rate of 0.35 mL/min, a column temperature of 40°C and an injection volume of 2 μL. The effluent was alternatively connected to an ESI‐triple quadrupole‐linear ion trap (QTRAP)‐MS. Metabolites were detected in MRM mode. De‐clustering potential (DP) and collision energy (CE) for individual MRM transitions were optimised. Specific MRM transitions were monitored for each time period based on the metabolites eluted during that period. MS data were processed and quantified using Analyst software (v1.6.3). Multivariate statistical analyses, including principal component analysis and orthogonal partial least squares discriminant analysis, as well as univariate statistics (fold change analysis and hypothesis testing), were conducted to identify differential metabolites. The threshold for DEMs was set at VIP ≥ 1 and |log_2_FC| ≥ 1 or *p* < 0.05.

### 4.16 KEGG Enrichment of Genes and Metabolites

For DEGs, B73_V4 names were converted to corresponding GeneIDs according to NCBI (https://ftp.ncbi.nlm.nih.gov/gene/DATA/GENE_INFO/Plants/Zea_mays.gene_info.gz). GeneIDs were subjected to KEGG analysis using KOBAS (http://kobas.cbi.pku.edu.cn/). For DEMs, compound names were converted to KEGG compound IDs using MetaboAnalyst (https://www.metaboanalyst.ca/) or CTS (http://cts.fiehnlab.ucdavis.edu/batch). All DEMs were subsequently analysed for KEGG analysis on the Metware cloud platform (https://cloud.metware.cn/#/tools). DEGs and DEMs in the same pathway were annotated by KEGG Mapper‐Colour (https://www.kegg.jp/kegg/mapper/color.html).

### 4.17 DX Recovery Experiment

*ZmTH1* knockout line (*ko*) was grown in 19 cm × 17 cm pots under greenhouse conditions with a 16 h light (25°C)/8 h dark (18°C) photoperiod. After germination, five seedlings per pot were maintained, and 20 mL of 0.1 g/L DX (ISOREAG) or water was applied every 5 days. For each application, DX was sprayed on the leaf surface, and the remainder was poured into the soil. Treatment continued until significant differences appeared between DX‐treated and untreated knockout plants. At this stage, leaves from *ko* plants (which were nearly dead) and green leaves from DX‐treated plants were collected for chlorophyll content measurement.

### 4.18 Abiotic Stress Treatments

B104 seeds were immersed in 0.1 g/L VB1 solution or double‐distilled water for 6 h, while *ZmTH1* overexpression lines (*OE1*, *OE3*, *OE4*) were immersed in double‐distilled water for 6 h. Seeds were subsequently planted in 8 cm × 8 cm pots in the greenhouse, with three pots allocated per abiotic stress treatment. Following germination, 9–10 seedlings were maintained per pot. Upon the emergence of the third leaf, plants received either 0.1 g/L VB1 treatment for B104 or double‐distilled water for *OE* and WT. Two days later, plants underwent various abiotic stress treatments. For salt and alkaline stress, pots were saturated with 100 mM NaCl or 50 mM Na_2_CO_3_ solution. For cold stress, pots were maintained in a 4°C chamber for 1 week, followed by greenhouse recovery. For heat stress, pots were placed in an incubator at 16 h light (42°C)/8 h dark (35°C) for 1 week, followed by greenhouse recovery. For drought stress, irrigation was suspended until visible symptoms manifested, followed by rewatering. For flood stress, pots were submerged to the stem base level until visible symptoms appeared, followed by drainage. MDA Content Assay Kit (Solarbio) was utilised to determine MDA content in leaves.

### 4.19 Statistical Analysis and Visualisation

Statistical analyses were conducted using R (v4.3.2). The chi‐squared test was employed for the *χ* ^2^ test, and *t_test* for Student's *t*‐test. Correlation analysis was performed using the *cor* function. Heatmaps were generated using pheatmap (v1.0.12), and principal component analysis was performed using PCAtools (v2.14.0). Data preprocessing, visualisation, multiple comparisons and statistical analyses were performed using the following R packages: tidyverse (v2.0.0), reshape2 (v1.4.4), ggplot2 (v3.5.1), ggpubr (v0.6.0), rstatix (v0.7.2), agricolae (v1.3.7) and export (v0.3.0).

## Author Contributions

T.Z., Z.Z., and H.C. planned and designed the research. T.Z. performed the experiments, analysed the data, and drafted the manuscript. J.Z., B.Y., and Q.W. helped with the experiments of heterologous expression, plant materials treatment and transmission electron microscopy. J.Y., P.X., and J.C. conducted the measurement of TMP, VB1, and TDP content. H.C. and Z.Z. revised the manuscript.

## Conflicts of Interest

The authors declare no conflicts of interest.

## Supporting information


**Figure S1:** 1 bp deletion in *Zm00001d041829* of *pldg1* and the ASP marker.
**Figure S2:** VB1 supplementation restored *pldg1* in adults.
**Figure S3:** Phylogenetic analysis of *ZmTH1* homologues in prokaryotes and eukaryotes.
**Figure S4:** Zm00001d035329 shared 94.6% identity within TMP‐S domain of *ZmTH1*.
**Figure S5:** Confirmation after *thib*, *thid* and *thie* knockout in *E.coli* MG1655 strain.
**Figure S6:** VB1 addition to M9 medium restored the growth of all transgenic strains.
**Figure S7:** Significantly (*p* < 0.05) up‐regulated (A) and down‐regulated (B) pathways of differentially expressed genes.
**Figure S8:** Differentially expressed metabolites in pathways related to TDP‐dependent enzymes.
**Figure S9:** Pathway of carbon fixation in photosynthetic organisms and significantly changed genes/metabolites.
**Figure S10:** Pathway of pyruvate metabolism and significantly changed genes/metabolites.
**Figure S11:** BCAA degradation pathway and significantly changed genes/metabolites.
**Figure S12:** Pentose phosphate pathway and significantly changed genes/metabolites.
**Figure S13:** BCAA synthesis pathway and significantly changed genes/metabolites.
**Figure S14:** Pathway of citrate cycle and significantly changed genes/metabolites.
**Figure S15:** Methylerythritol phosphate pathway and significantly changed genes/metabolites in the synthesis pathway of terpene‐related compound.
**Figure S16:** Photophosphorylation pathway and significantly changed genes.
**Figure S17:** Oxidative phosphorylation pathway and significantly changed genes.
**Figure S18:** qRT‐PCR validation of genes in RNA‐seq.
**Figure S19:** *ZmTH1* expression between *ko*, *OE* and WT materials.
**Figure S20:** Structural prediction of *ZmTH1* before and after mutation.
**Figure S21:** Phenotypes of the *ZmTH1* knockout line before and after DX supplementation.

**Table S1:** Genes in the 1.4 Mb interval.
**Table S2:** Primers and their usages in this work.
**Table S3:** Gene function and RNA‐seq data of DEGs in Figure S9–S17.
**Table S4:** Genes used in RNA‐seq confirmation and their primers.

## Data Availability

Clean RNA‐seq data reported in this article have been uploaded to the Genome Sequence Archive in National Genomics Data Center under accession number CRA021719 (https://bigd.big.ac.cn/gsa/browse/CRA021719). Accession Numbers: Sequences can be found in the MaizeGDB (https://www.maizegdb.org/) or EcoCyc (https://ecocyc.org/) data libraries under accession number *ZmTH1* (*Zm00001d041829*), *ZmTMPS1* (*Zm00001d035329*), *thiD* (UniProt: P76422), *thiE* (UniProt: P30137).
