# Supplementary material for: ZmTH1 Is Vital for Healthy Plant Growth and Promotes Cold/Drought Tolerance by Regulating Thiamin Diphosphate‐Dependent Metabolisms in Maize
Source: Plant Biotechnol J. 2025 Oct 13;24(3):1234–50. doi: 10.1111/pbi.70400 (PMC12946460; doi:10.1111/pbi.70400)
Supplement: Supplementary file 1 — Figure S1: 1 bp deletion in Zm00001d041829 of pldg1 and the ASP marker. Figure S2: VB1 supplementation restored pldg1 in adults. Figure S3: Phylogenetic analysis of ZmTH1 homologues in prokaryotes and eukaryotes. Figure S4: Zm00001d035329 shared 94.6% identity within TMP‐S domain of ZmTH1. Figure S5: Confirmation after thib, thid and thie knockout in E.coli MG1655 strain. Figure S6: VB1 addition to M9 medium restored the growth of all transgenic strains. Figure S7: Significantly (p < 0.05) up‐regulated (A) and down‐regulated (B) pathways of differentially expressed genes. Figure S8: Differentially expressed metabolites in pathways related to TDP‐dependent enzymes. Figure S9: Pathway of carbon fixation in photosynthetic organisms and significantly changed genes/metabolites. Figure S10: Pathway of pyruvate metabolism and significantly changed genes/metabolites. Figure S11: BCAA degradation pathway and significantly changed genes/metabolites. Figure S12: Pentose phosphate pathway and significantly changed genes/metabolites. Figure S13: BCAA synthesis pathway and significantly changed genes/metabolites. Figure S14: Pathway of citrate cycle and significantly changed genes/metabolites. Figure S15: Methylerythritol phosphate pathway and significantly changed genes/metabolites in the synthesis pathway of terpene‐related compound. Figure S16: Photophosphorylation pathway and significantly changed genes. Figure S17: Oxidative phosphorylation pathway and significantly changed genes. Figure S18: qRT‐PCR validation of genes in RNA‐seq. Figure S19: ZmTH1 expression between ko, OE and WT materials. Figure S20: Structural prediction of ZmTH1 before and after mutation. Figure S21: Phenotypes of the ZmTH1 knockout line before and after DX supplementation. [file PBI-24-1234-s002.docx]

**Supplemental information**


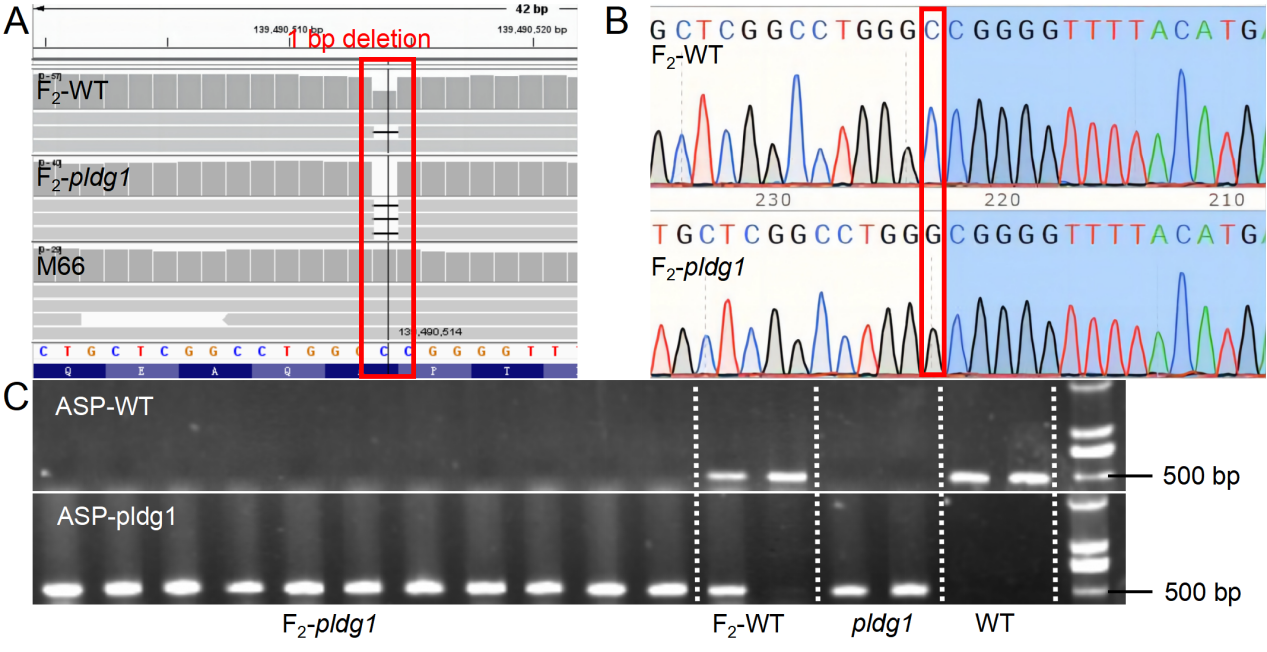


**Figure S1. 1 bp deletion in *Zm00001d041829* of *pldg1* and the ASP marker**

**(A)** Exhibition of the 1-bp deletion (red box) in *Zm00001d041829* of BAM file. The gray bar indicates 1 bp, and the black horizontal line indicates 1 bp deletion. The wild-type parent M66 had no deletion. All *pldg1* individuals in F_2_ deleted 1 bp, and the WT individuals in F_2_ had both genotypes. **(B)** Sequencing revealed the 1 bp deletion in *pldg1*. **(C)** ASP marker was exploited for 1 bp deletion. WT bands could only be amplified by ASP-WT and mutant bands could only be amplified by ASP-pldg1.


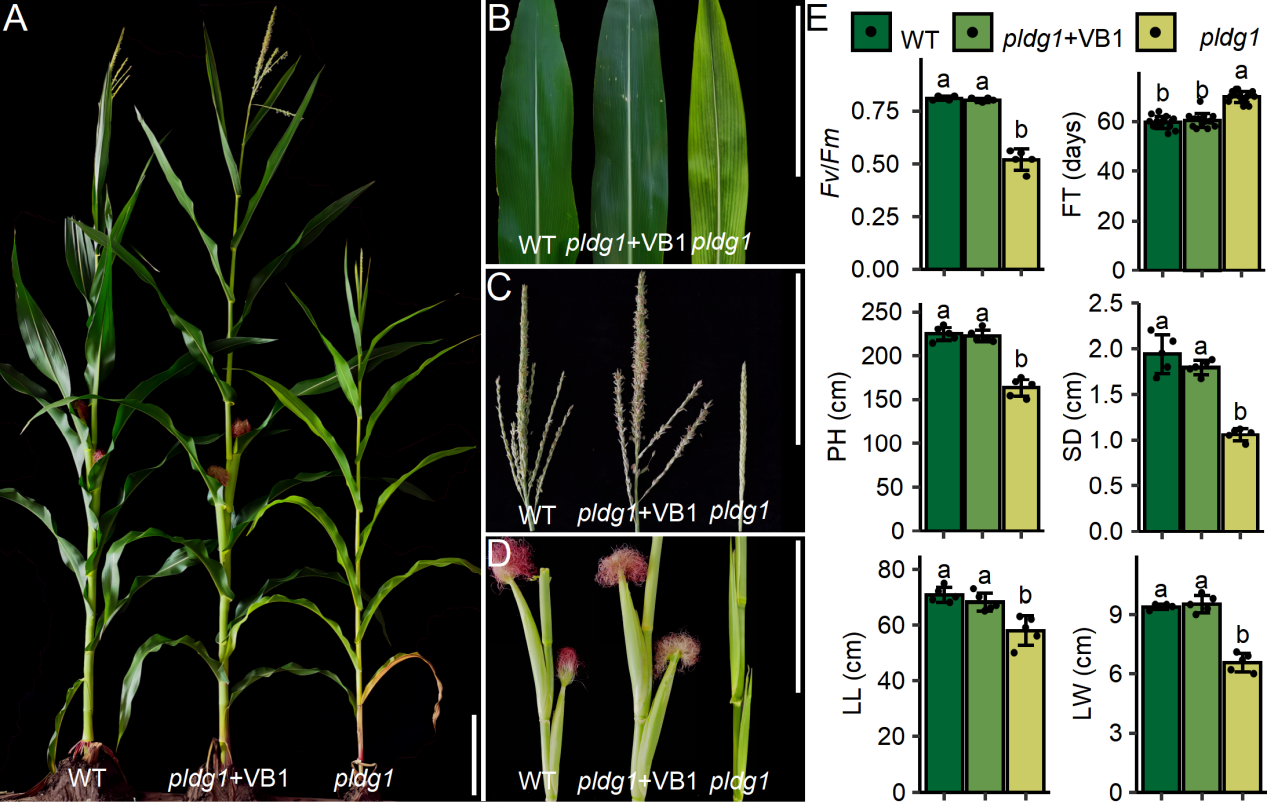


**Figure S2. VB1 supplementation restored *pldg1* in adults**

1. **D)** Behaviors of *pldg1* in adults before and after VB1 supplementation. **(E)** VB1 supplementation recovered the *Fv*/*Fm*, flowering time (FT), plant height (PH), stem diameter(SD), leaf length (LL), leaf width (LW) of *pldg1* to WT level. Values are means ± standard deviation (SD). Different letters show significant difference (One-way ANOVA, LSD.test. *p* < 0.01).


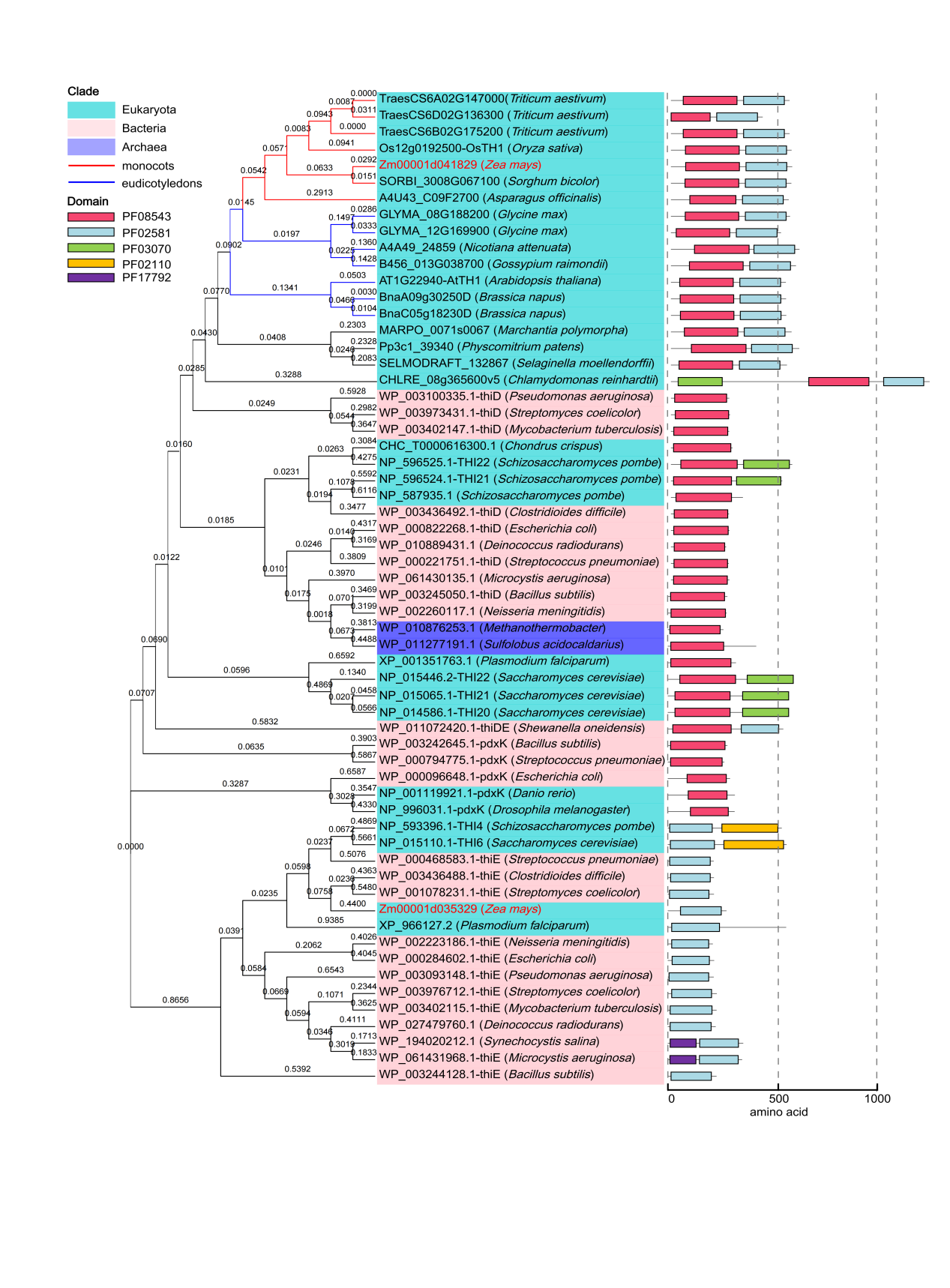


**Figure S3. Phylogenetic analysis of ZmTH1 homologs in prokaryotes and eukaryotes**

ZmTH1 contains two conserved domains, HMPP-K and TMP-S, they are mostly encoded separately by *thiD* and *thiE* in bacteria, they combine with other domains separately in yeast, they resemble ZmTH1 in other plants, while no homologs are found in human and mice. PF08543 is HMPP-K domain, PF02581 is TMP-S domain, PF03070 is TENA_THI-4 family domain, PF02110 is a domain containing thiM function, PF17792 is a thiD2 domain similar to HMPP-K domain. Number on each branch is the degree of genetic variation, a smaller number indicates a closer evolutionary distance. Zm00001d041829 (ZmTH1) and Zm00001d035329 (ZmTMPS1) are highlighted in red fonts.


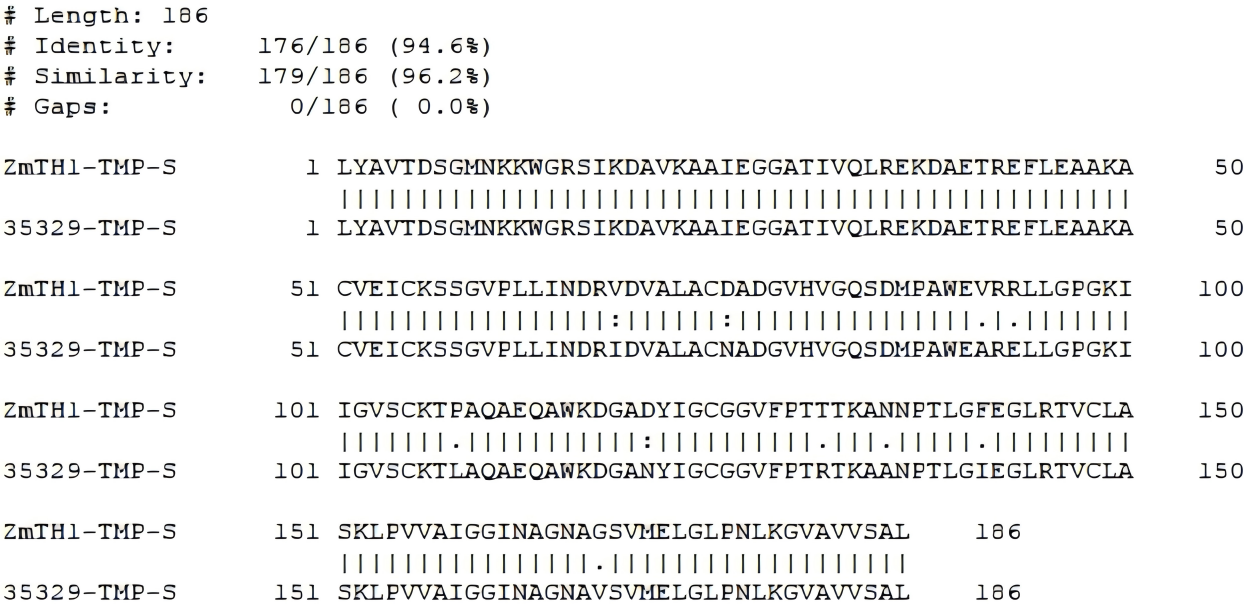


**Figure S4. Zm00001d035329 shared 94.6% identity within TMP-S domain of ZmTH1**

“Identity” and “Similarity” represents sequence consistency and sequence similarity, respectively.


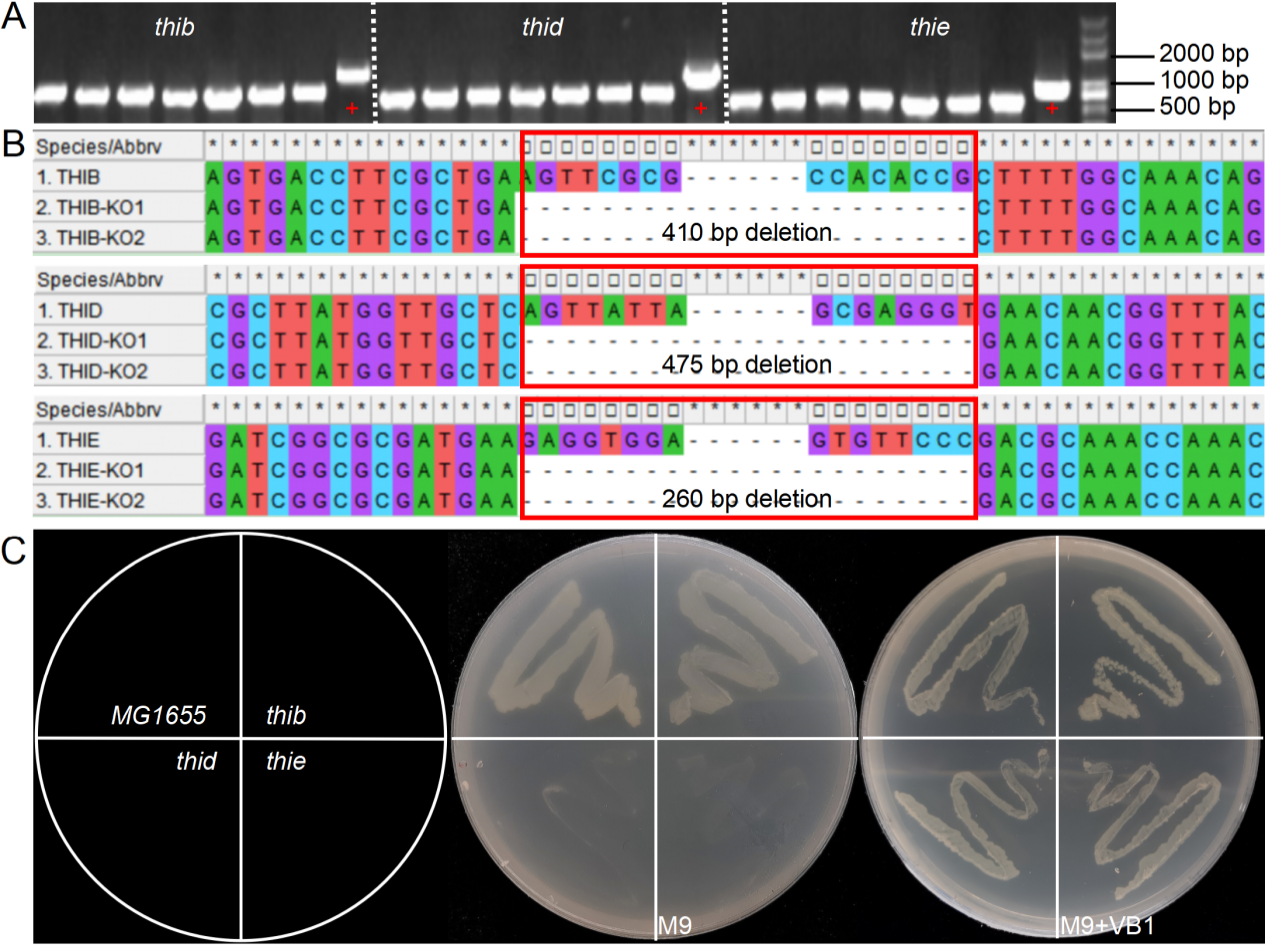


**Figure S5. Verification of *thib*, *thid*, and *thie* knockout in *E. coli* MG1655**

**(A)** PCR detection after *thib*, *thid* and *thie* knockout. "+" indicates the wild type. **(B)** Sequencing results after *thib*, *thid* and *thie* knockout. Red box indicates the missing fragment length after knockout compared with their wild type. **(C)** Growth confirmation of *thib*, *thid* and *thie* knockout strains and the wild type MG1655 strain in M9 medium with/without VB1. *thid* and *thie* could not grow on M9 medium without VB1, but could be rescued after VB1 addition. Defective *E.coli* strains NI400 was used in *Arabidopsis* (Ajjawi et al., 2007a), but its mutation was likely in *thiB* rather than *thiE* as shown in the database (https://cgsc2.biology.yale.edu/). However, genomic annotation indicates that *thiE* encodes TMP-S domain. Therefore, we also performed a knockout of *thiB* to confirm that.


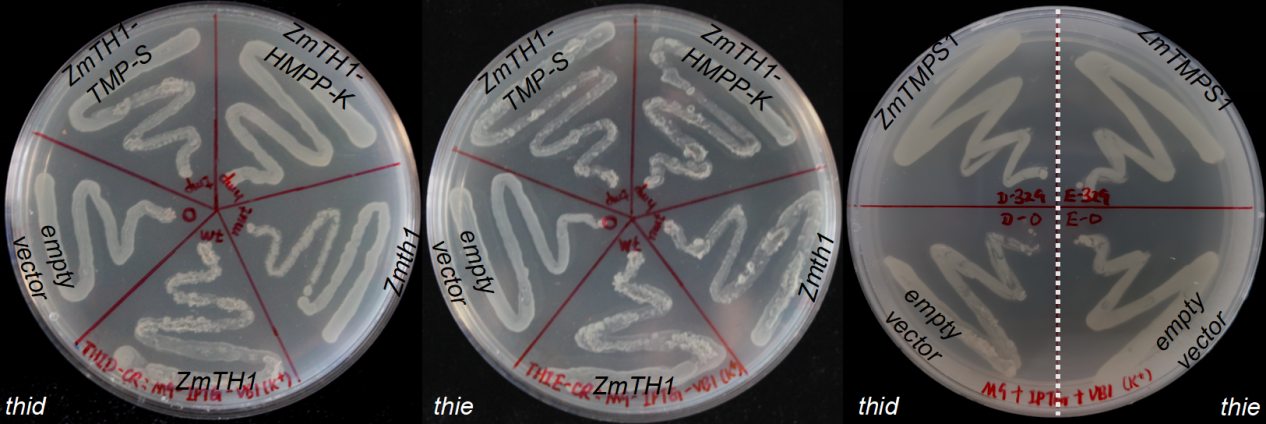


**Figure S6. VB1 addition to M9 medium restored the growth of all transgenic strains**

*ZmTH1-HMPP-K* and *ZmTH1-TMP-S* represent HMPP-K domain coding sequence and TMP-S domain coding sequence of *ZmTH1*, respectively.


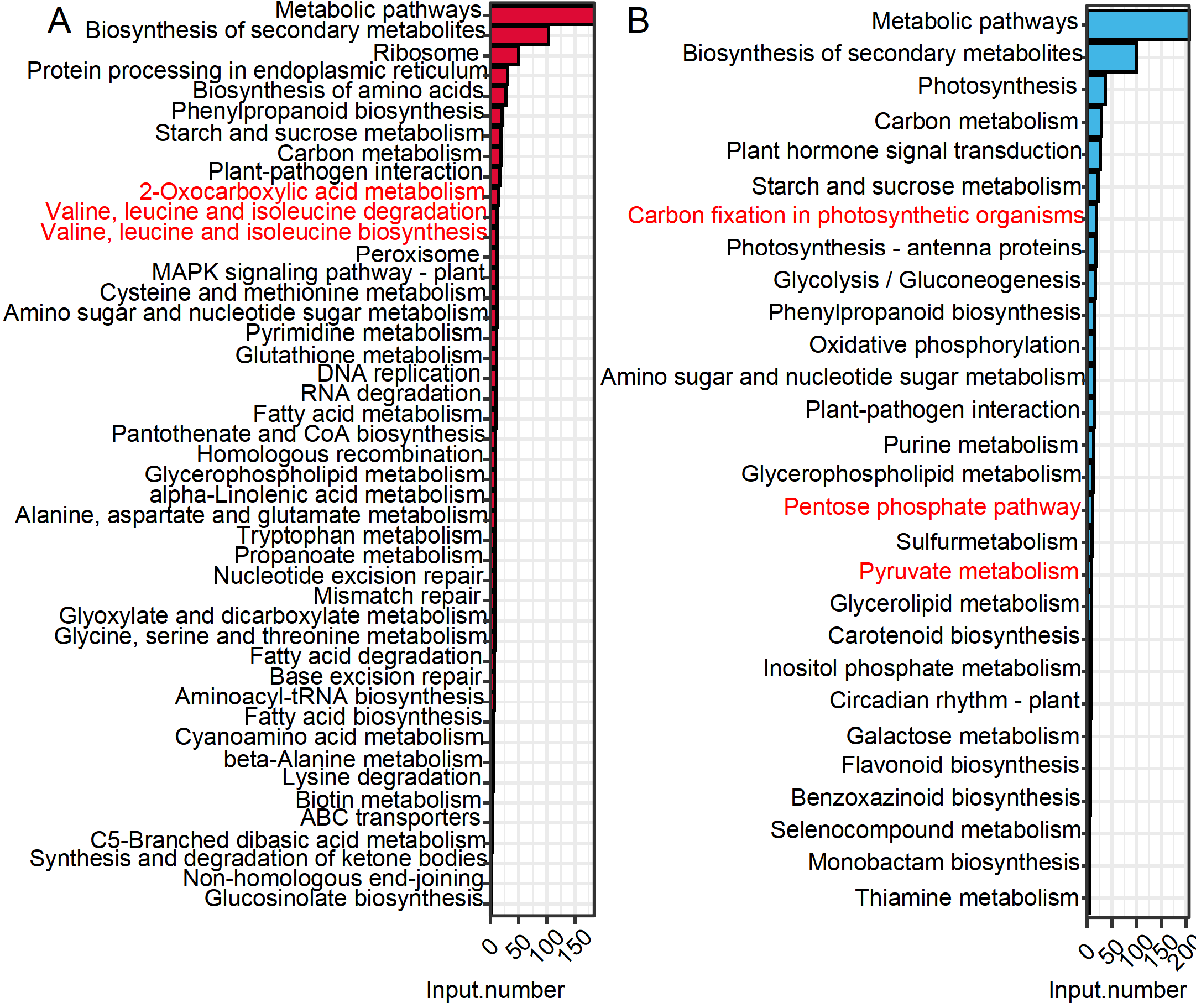


**Figure S7. Significantly (*p*< 0.05) up-regulated (A) and down-regulated (B) pathways of differentially expressed genes**

“Input.number” indicates the number of differentially expressed genes in each pathway. Red fonts indicate pathways requiring TDP-dependent enzymes directly.


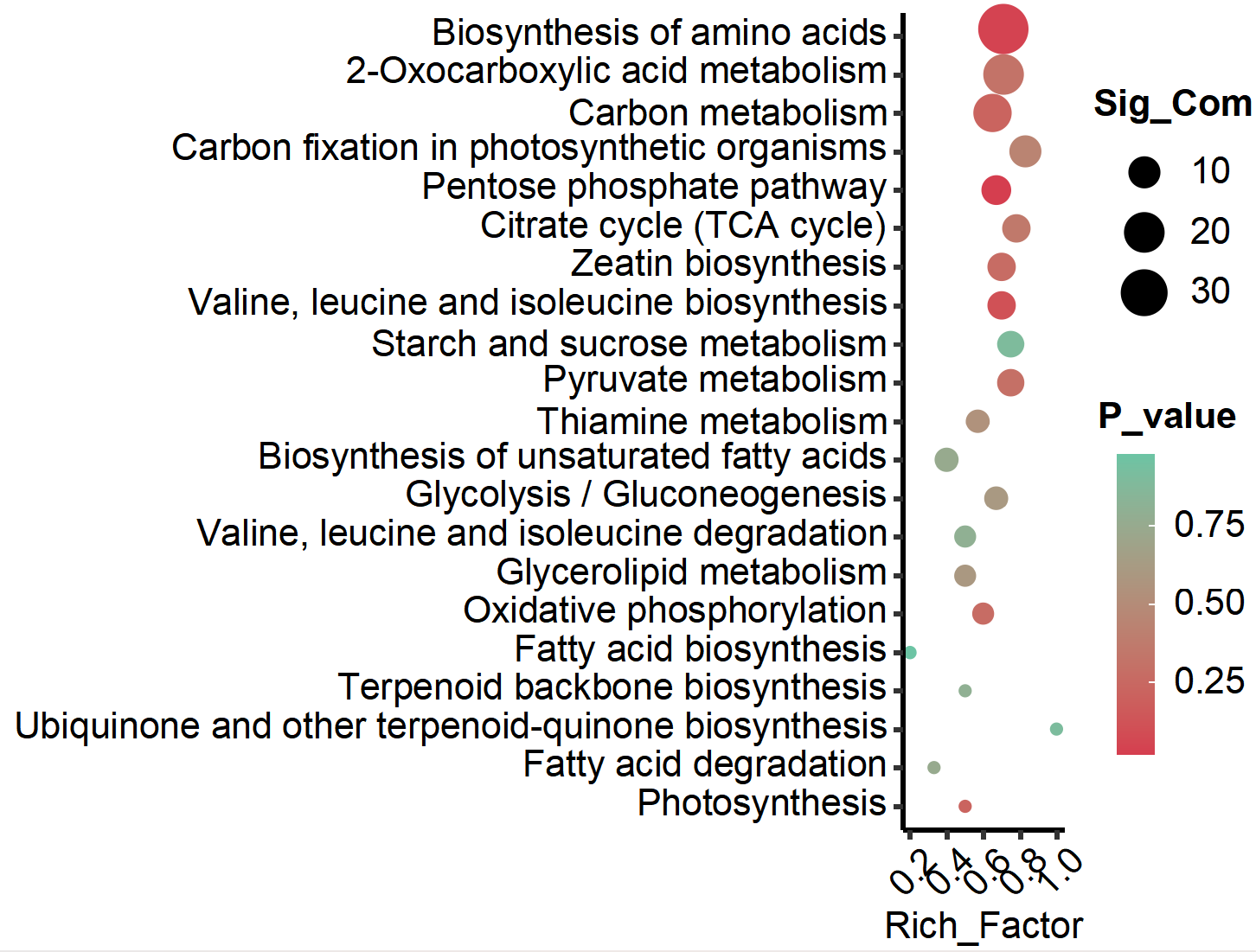


**Figure S8. Differentially expressed metabolites in pathways related to TDP-dependent enzymes**

Sig_Com: number of metabolites with significant change. Rich_factor: ratio of the number of differentially expressed metabolites in a given pathway to the total number of metabolites annotated in that pathway, a higher value signifies a greater enrichment.


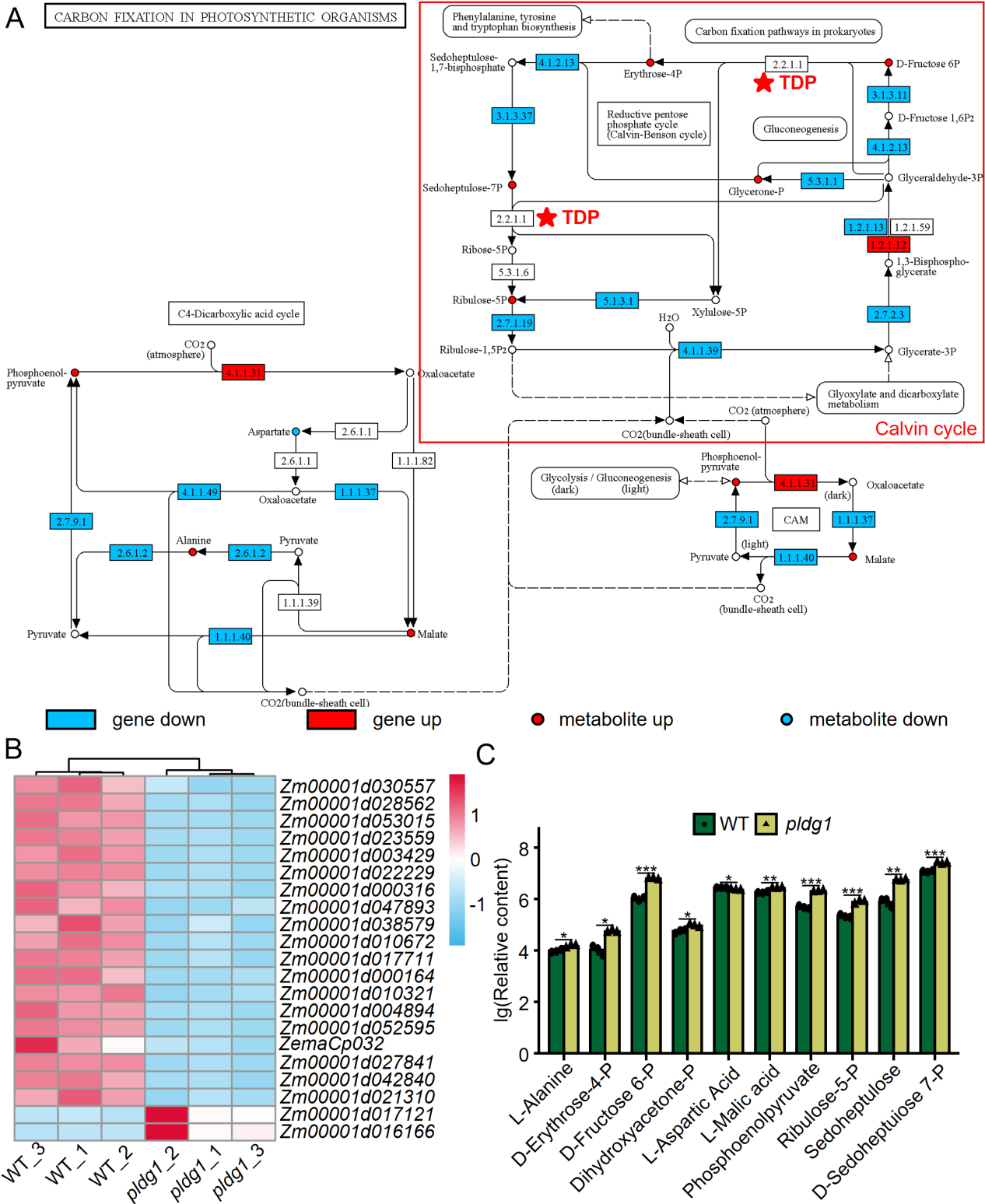


**Figure S9. Pathway of carbon fixation in photosynthetic organisms and significantly changed genes/metabolites**

**(A)** Pathway of carbon fixation in photosynthetic organisms. Red box marks the Calvin cycle pathway. ☆TDP indicates the enzyme needs TDP as a cofactor. Up/down-regulation of genes/metabolites is the change of *pldg1* relative to WT. **(B, C)** are the gene/metabolite significantly changed in (A). *: *p* < 0.05. **, ***: *p* < 0.01.


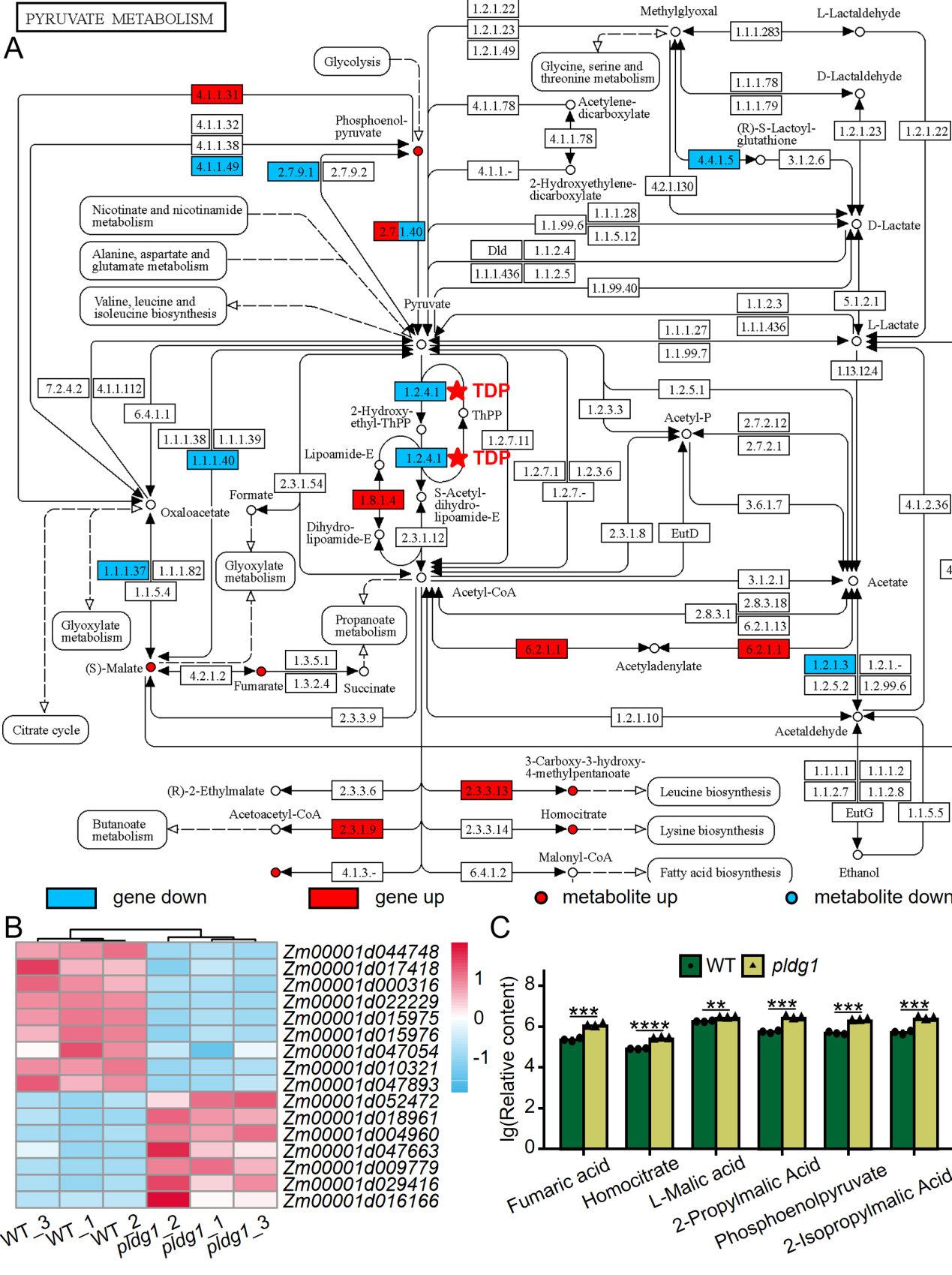


**Figure S10. Pathway of pyruvate metabolism and significantly changed genes/metabolites**

**(A)** Pathway of pyruvate metabolism. ☆TDP indicates the enzyme needs TDP as a cofactor. Up/down-regulation of genes/metabolites is the change of *pldg1* relative to WT. **(B, C)** are the gene/metabolite significantly changed in (A). **, ***, ****: *p* < 0.01.


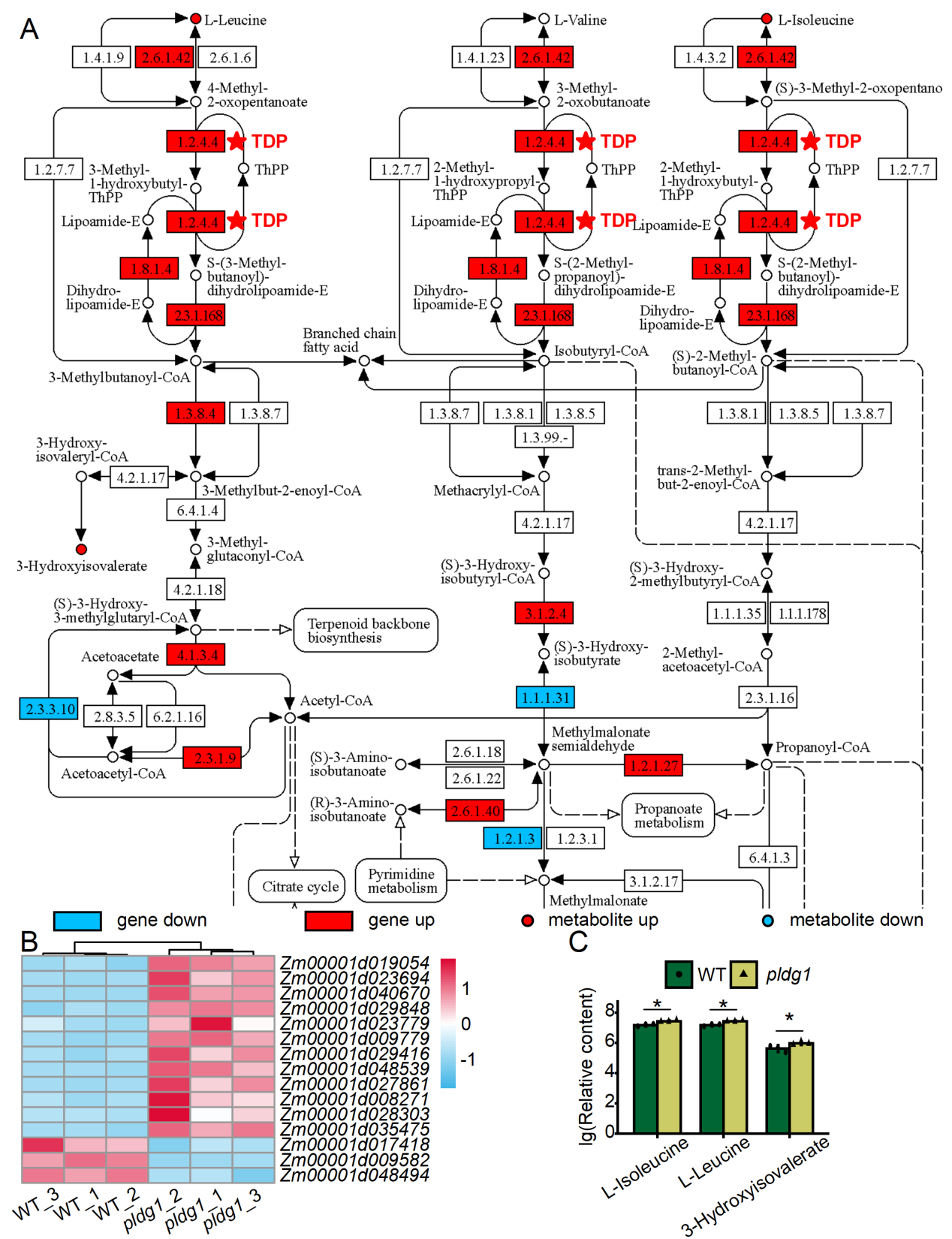


**Figure S11. BCAA degradation pathway and significantly changed genes/metabolites**

**(A)** BCAA degradation pathway. ☆TDP indicates the enzyme needs TDP as a cofactor. Up/down-regulation of genes/metabolites is the change of *pldg1* relative to WT. **(B, C)** are gene/metabolite significantly changed in (A). *: *p* < 0.05.


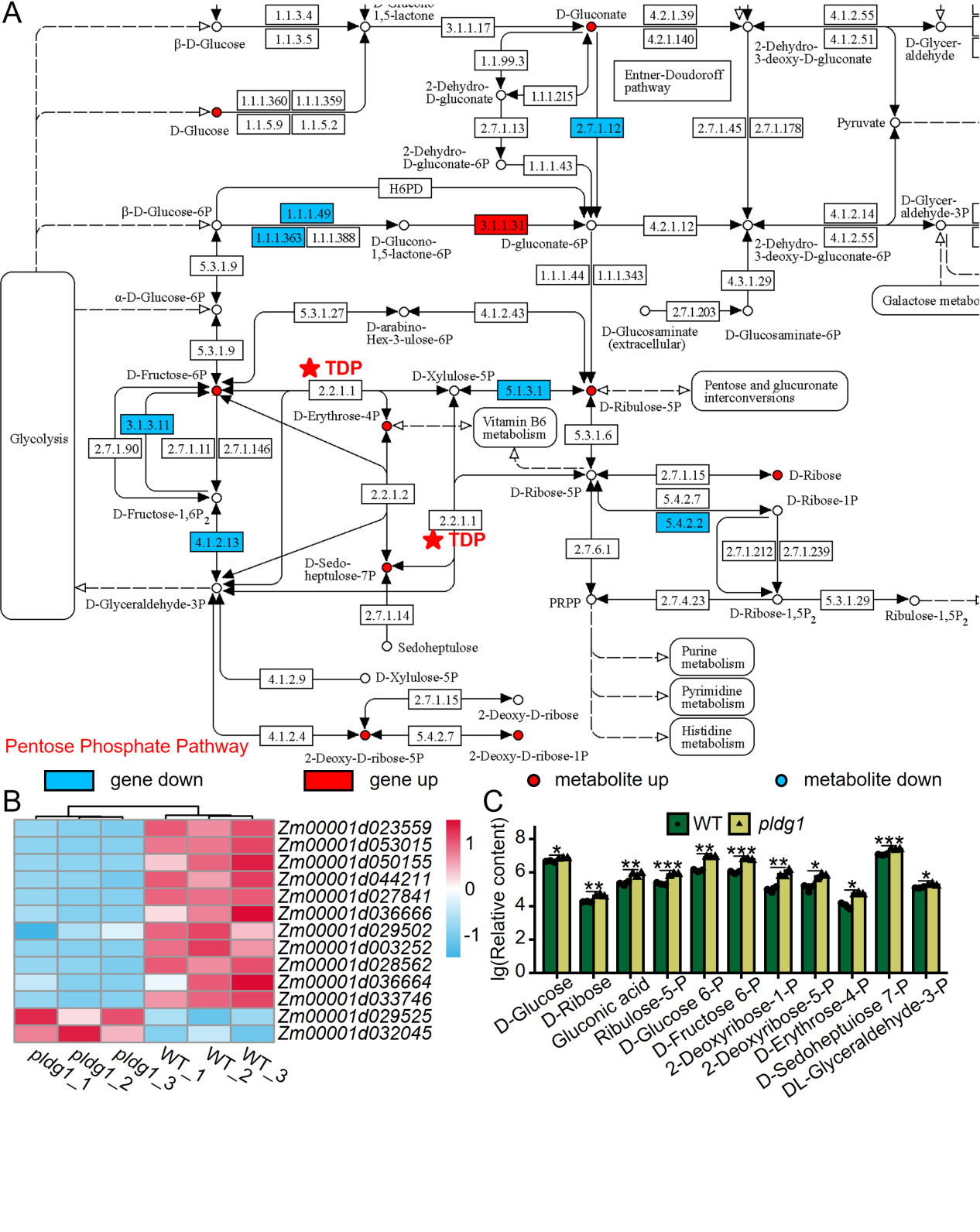


**Figure S12. Pentose phosphate pathway and significantly changed genes/metabolites**

**(A)** Pentose phosphate pathway. ☆TDP indicates the enzyme needs TDP as a cofactor. Up/down-regulation of genes/metabolites is the change of *pldg1* relative to WT. **(B, C)** are gene/metabolite significantly changed in (A). *: *p* < 0.05. **, ***: *p* < 0.01.


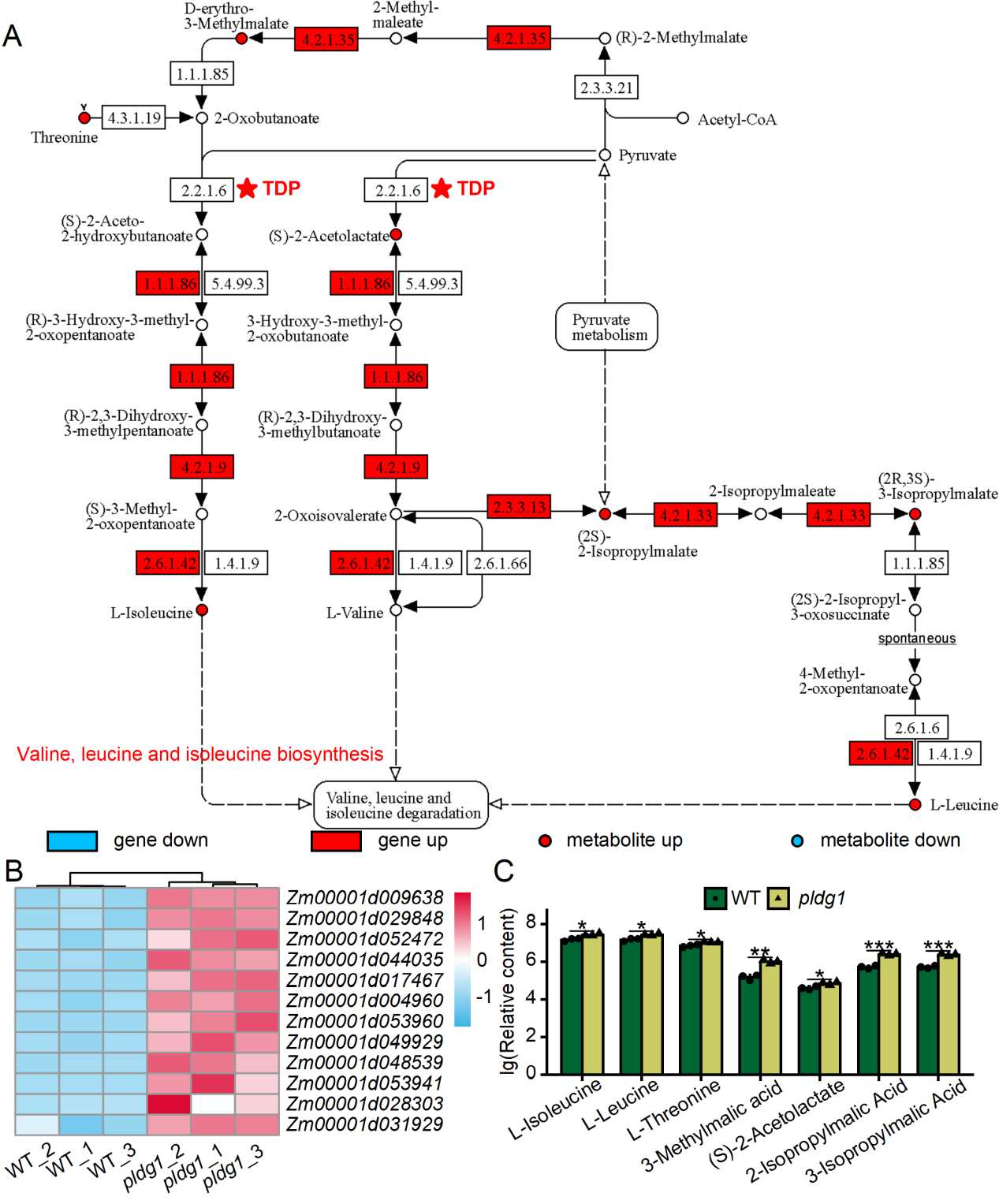


**Figure S13. BCAA synthesis pathway and significantly changed genes/metabolites**

**(A)** BCAA synthesis pathway. ☆TDP indicates that the enzyme needs TDP as a cofactor. Up/down-regulation of genes/metabolites is the change of *pldg1* relative to WT. **(B, C)** are the gene/metabolite significantly changed in (A) *: *p* < 0.05. **, ***: *p* < 0.01.


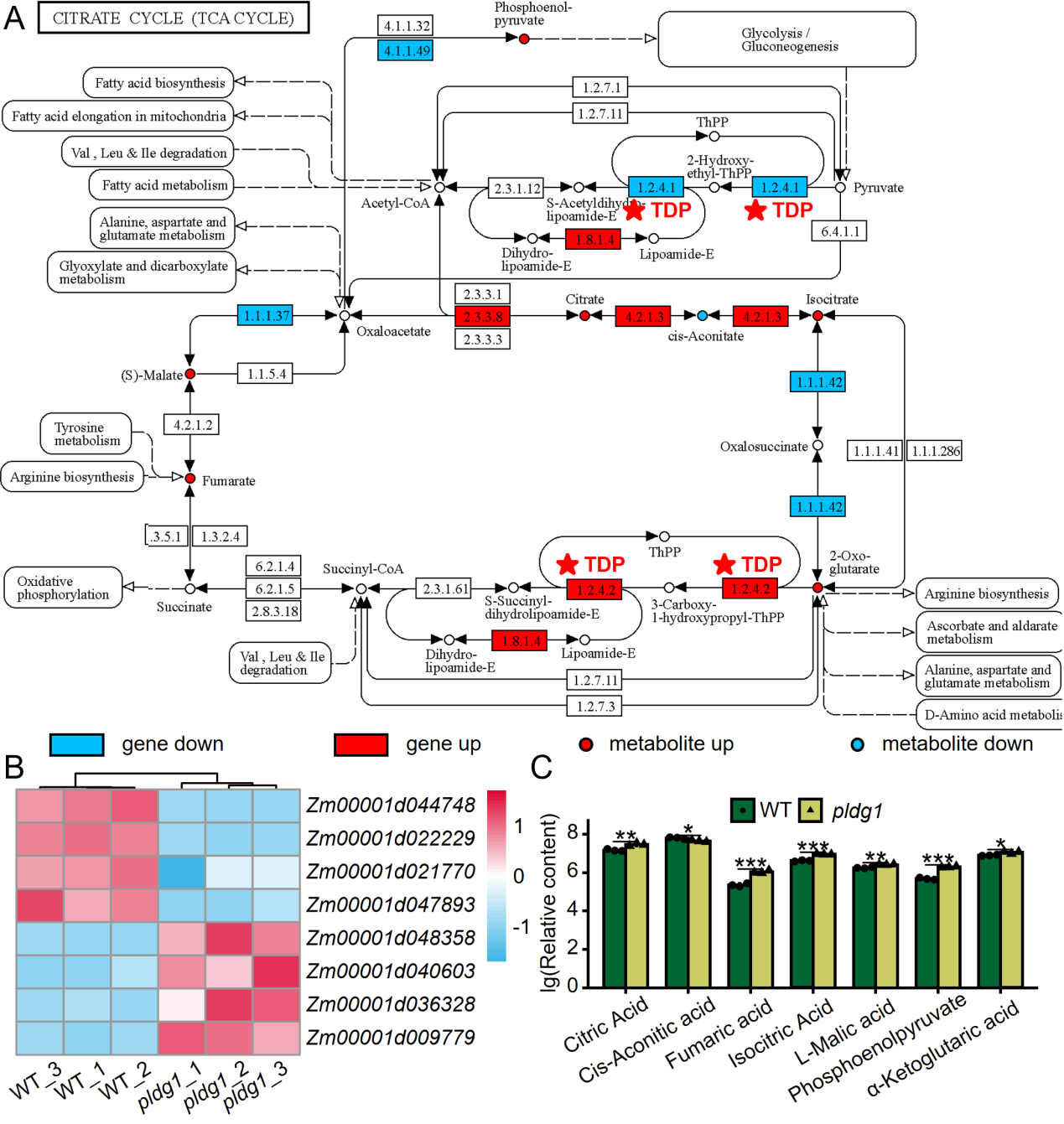


**Figure S14. Pathway of citrate cycle and significantly changed genes/metabolites**

**(A)** Pathway of citrate cycle. ☆TDP indicates that the enzyme needs TDP as a cofactor. Up/down-regulation of genes/metabolites is the change of *pldg1* relative to WT. **(B, C)** are gene/metabolite significantly changed in (A). *: *p* < 0.05. **, ***: *p* < 0.01.


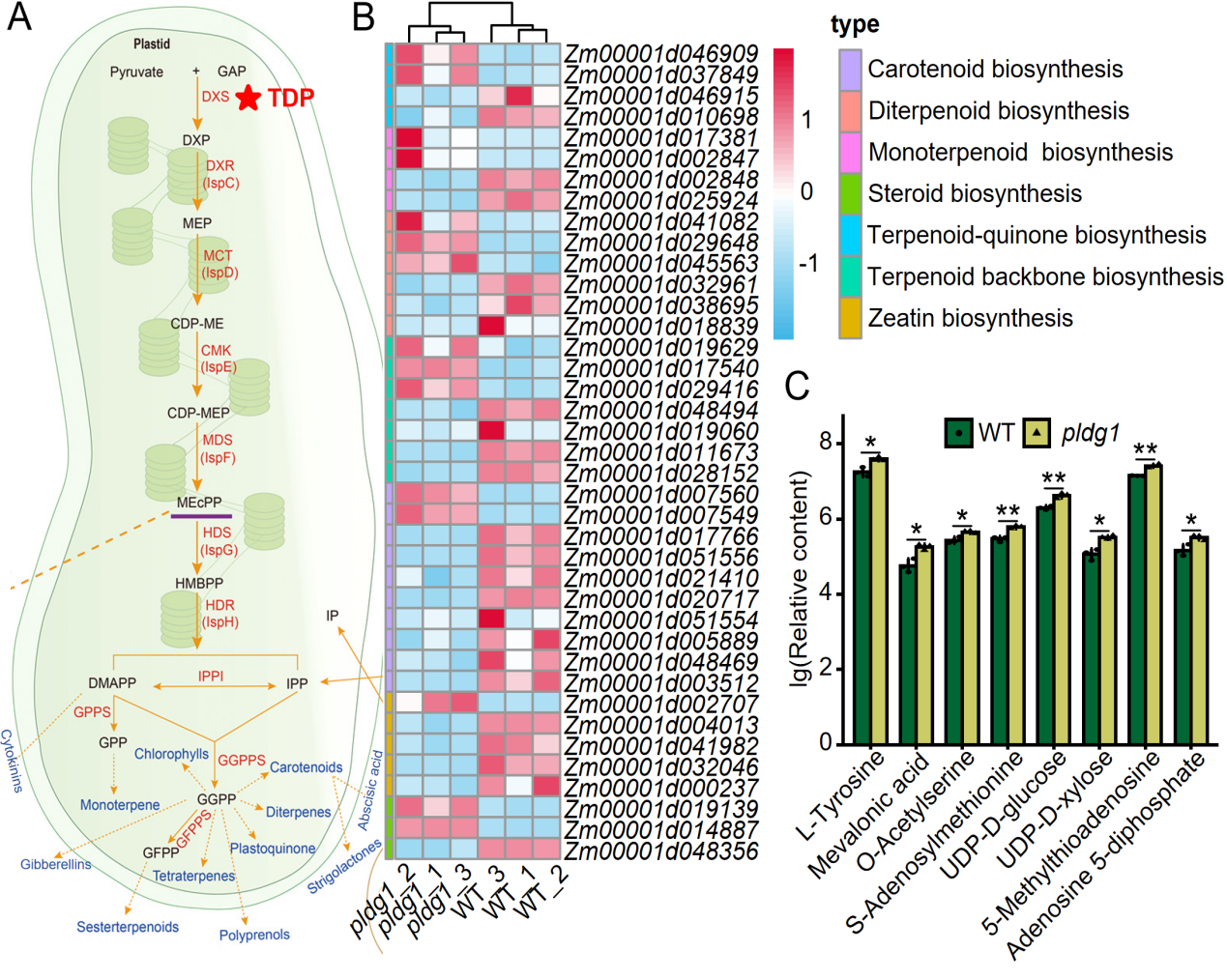


**Figure S15. Methylerythritol phosphate pathway and significantly changed genes/metabolites in the synthesis pathway of terpene-related compounds**

**(A)** Destination of terpenes synthesized by methylerythritol phosphate pathway (Pu et al., 2021). ☆TDP indicates that the enzyme needs TDP as a cofactor. DMAPP and IPP are bases for the synthesis of terpene-related compounds. **(B, C)** are genes/metabolites with significant changes in the synthesis pathway of terpene-related compounds. Up/down-regulation of genes/metabolites is the change of *pldg1* relative to WT. *: *p* < 0.05. **: *p* < 0.01.


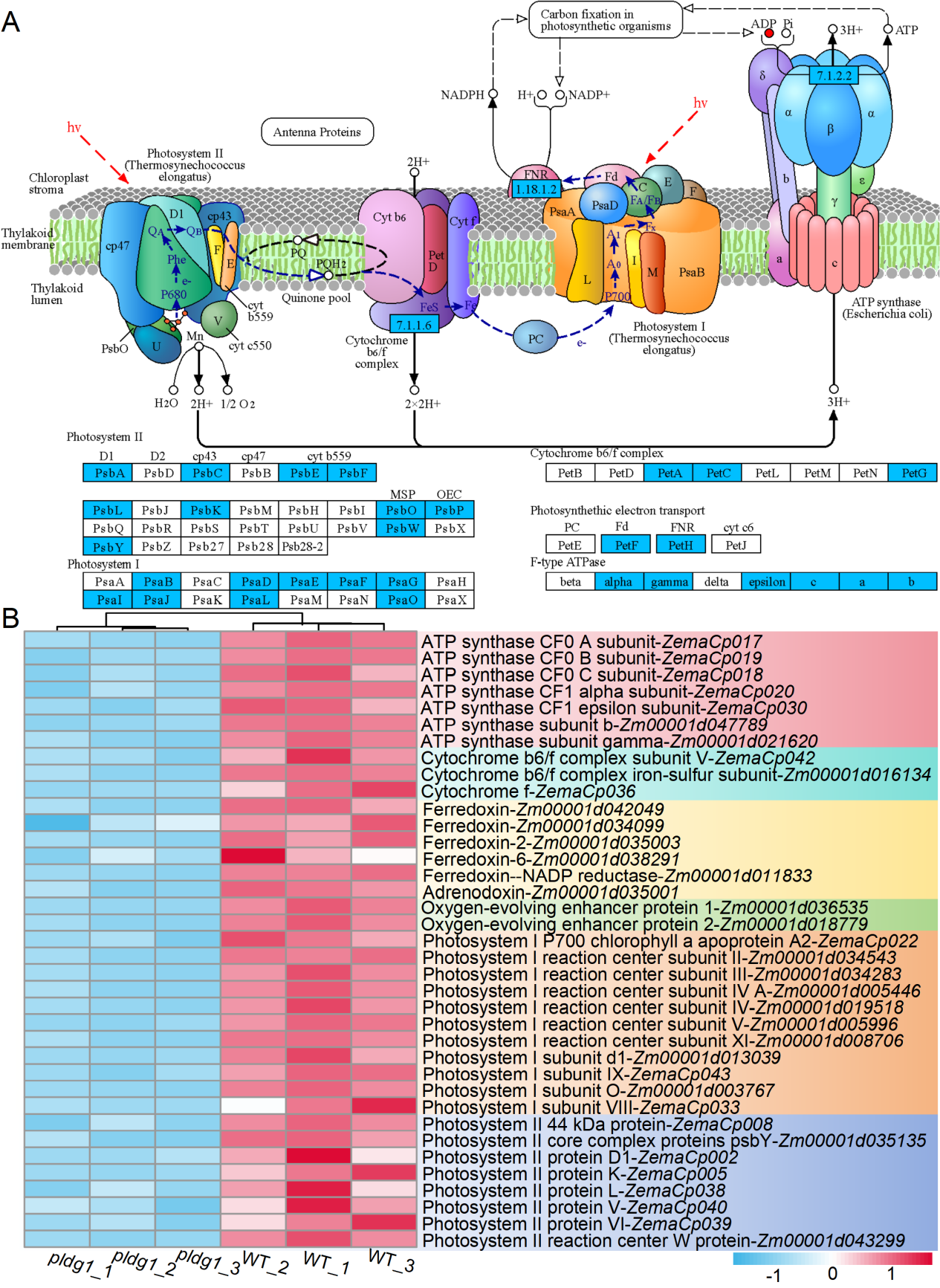


**Figure S16. Photophosphorylation pathway and significantly changed genes**

**(A)** Photophosphorylation pathway. Blue box shows significantly down-regulated genes. **(B)** are the genes with significant changes. Different background colors indicate different classifications. Up/down-regulation of genes is the change of *pldg1* relative to WT.


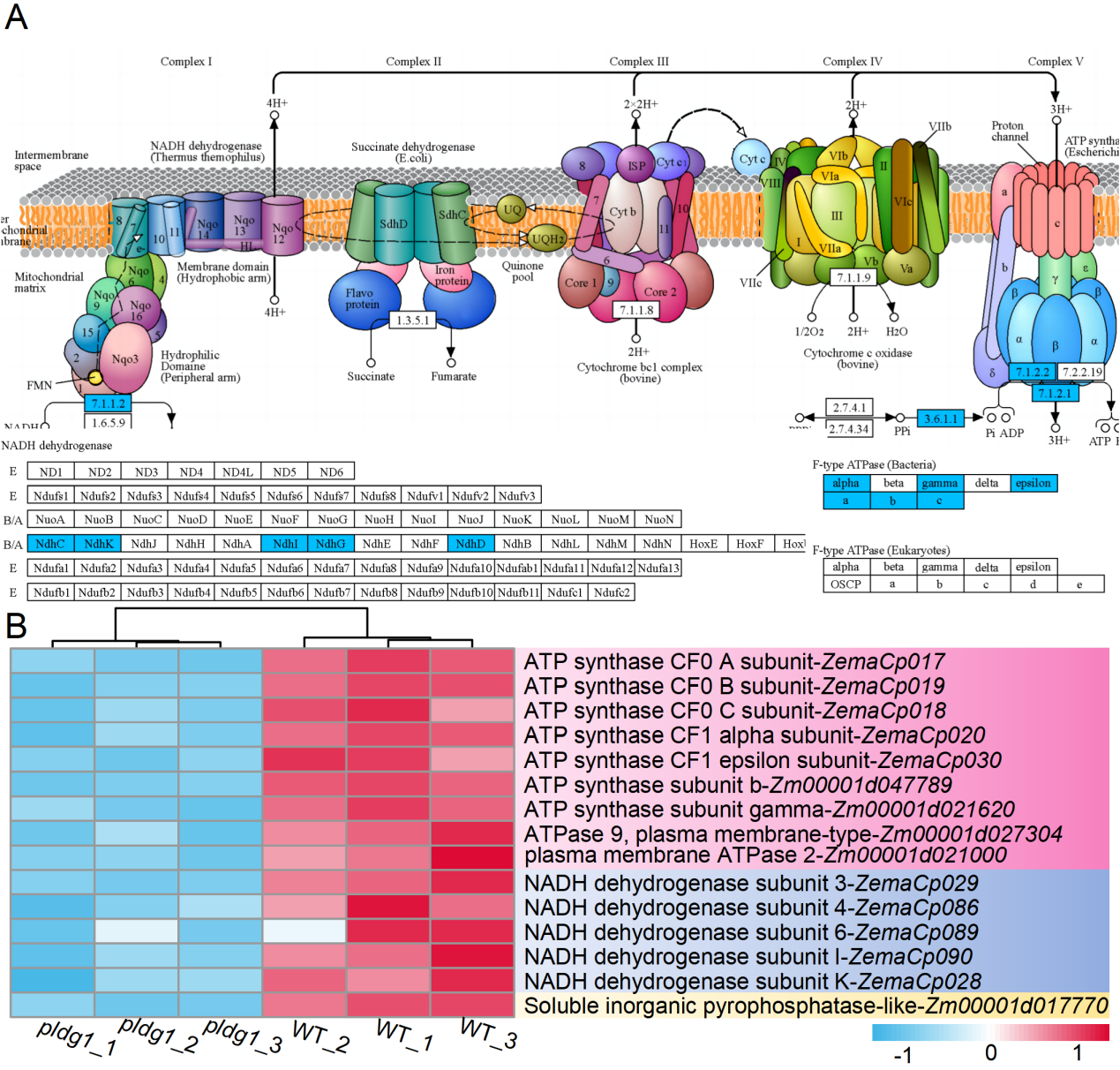


**Figure S17. Oxidative phosphorylation pathway and significantly changed genes**

**(A)** Oxidative phosphorylation pathway. Blue box shows significantly down-regulated genes. **(B)** are the genes with significant changes. Different background colors indicate different classifications. Up/down-regulation of genes is the change of *pldg1* relative to WT.


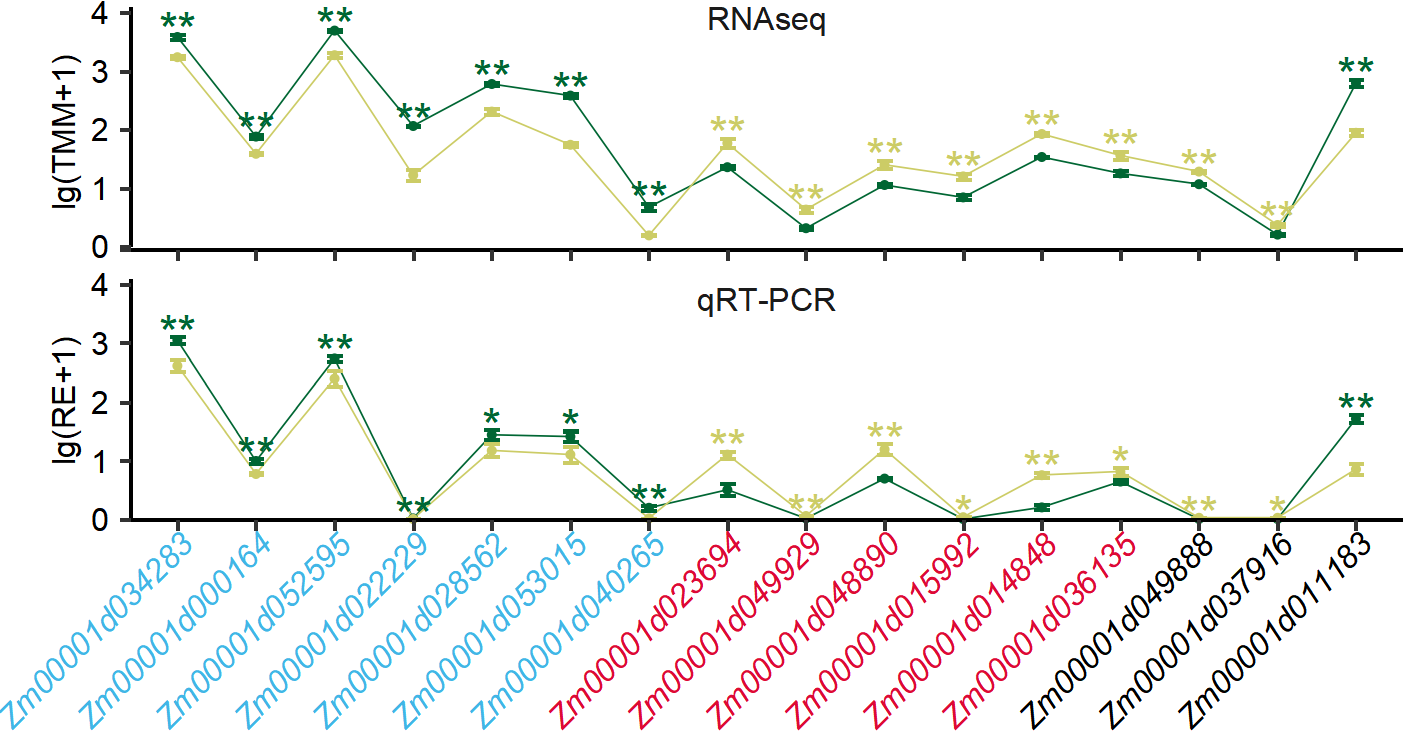


**Figure S18. qRT-PCR validation of genes in RNA-seq**

Blue/red fonts are significantly down/up regulated genes in *pldg1*, respectively. Black fonts are VB1 synthetic genes. TMM: trimmed mean of M-values. RE: relative expression. Values are means ± standard deviation (SD). *: *p* < 0.05. **: *p* < 0.01.


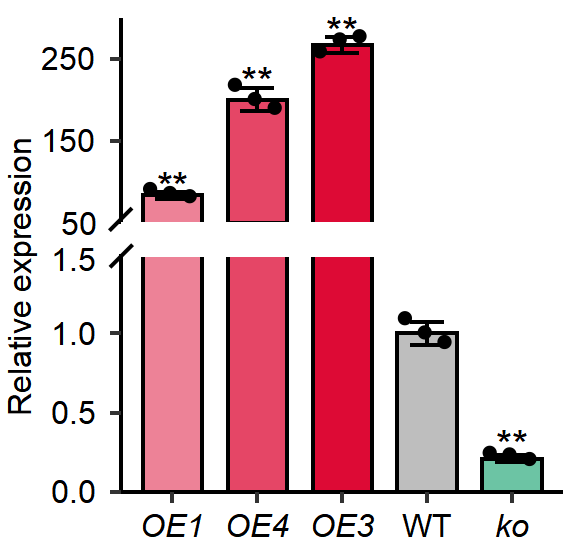


**Figure S19. *ZmTH1* expression between *ko*, *OE* and WT materials**

*OE*: *overexpression*. *ko*: *knockout*. WT: wild type. Values are means ± standard deviation (SD). n = 3. **: *p* < 0.01. Student’s *t*-test.


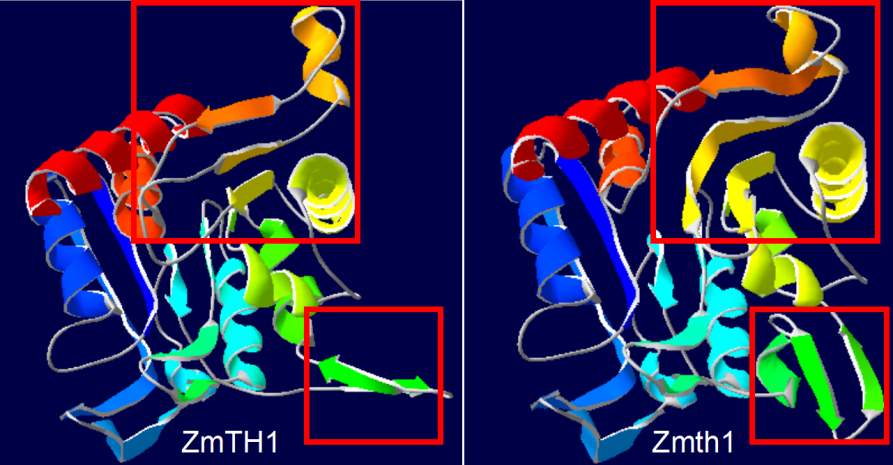


**Figure S20. Structural prediction of ZmTH1 before and after mutation**

Mutations of Zmth1 may alter the length and count of β-sheets in its protein secondary structure. Red box shows where the structure changed.


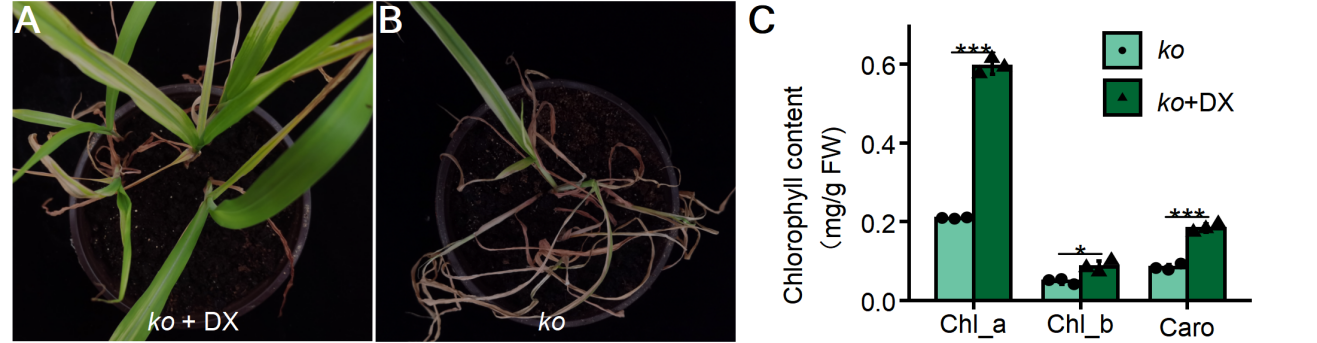


**Figure S21. Phenotypes of the *ZmTH1*** **knockout line before and after DX supplementation**

**(A, B)** Symptom of *ZmTH1* knockout line (*ko*) before and after DX supplementation. **(C)** Chlorophyll content of *ko* before and after DX supplementation. DX: 1-deoxy-D-xylulose, Chl_a: chlorophyll a, Chl_b: chlorophyll b, Caro: carotenoids. Values are means ± standard deviation (SD). n = 3. *: *p* < 0.05, ***: *p* < 0.01, Student’s *t*-test.
